# Transcriptional profiling sheds light on the fibrotic aspects of idiopathic subglottic tracheal stenosis

**DOI:** 10.3389/fcell.2024.1380902

**Published:** 2024-07-12

**Authors:** Martin Direder, Maria Laggner, Dragan Copic, Katharina Klas, Daniel Bormann, Thomas Schweiger, Konrad Hoetzenecker, Clemens Aigner, Hendrik Jan Ankersmit, Michael Mildner

**Affiliations:** ^1^ Laboratory for Cardiac and Thoracic Diagnosis, Regeneration and Applied Immunology, Department of Thoracic Surgery, Medical University of Vienna, Vienna, Austria; ^2^ Aposcience AG, Vienna, Austria; ^3^ Department of Orthopedics and Trauma-Surgery, Medical University of Vienna, Vienna, Austria; ^4^ Department of Internal Medicine III, Division of Nephrology and Dialysis, Medical University of Vienna, Vienna, Austria; ^5^ Department of Thoracic Surgery, Medical University of Vienna, Vienna, Austria; ^6^ Department of Dermatology, Medical University of Vienna, Vienna, Austria

**Keywords:** idiopathic subglottic stenosis, trachea, matrix, fibroblasts, plasma cells, Schwann cells, single cell RNA sequencing

## Abstract

Idiopathic subglottic stenosis (ISGS) is a rare fibrotic disease of the upper trachea with an unknown pathomechanism. It typically affects adult Caucasian female patients, leading to severe airway constrictions caused by progressive scar formation and inflammation with clinical symptoms of dyspnoea, stridor and potential changes to the voice. Endoscopic treatment frequently leads to recurrence, whereas surgical resection and reconstruction provides excellent long-term functional outcome. This study aimed to identify so far unrecognized pathologic aspects of ISGS using single cell RNA sequencing. Our scRNAseq analysis uncovered the cellular composition of the subglottic scar tissue, including the presence of a pathologic, profibrotic fibroblast subtype and the presence of Schwann cells in a profibrotic state. In addition, a pathology-associated increase of plasma cells was identified. Using extended bioinformatics analyses, we decoded pathology-associated changes of factors of the extracellular matrix. Our data identified ongoing fibrotic processes in ISGS and provide novel insights on the contribution of fibroblasts, Schwann cells and plasma cells to the pathogenesis of ISGS. This knowledge could impact the development of novel approaches for diagnosis and therapy of ISGS.

## 1 Introduction

Idiopathic subglottic stenosis (ISGS) is a rare pathology of the subglottic larynx and upper trachea, occurring at a rate of one in 400,000 per year (Gelbard et al., 2016a). It is characterized by fibrotic lesions with unknown etiology (Gelbard et al., 2016a). These lesions typically exhibit a circumferential pattern, extending up to a height of 5 cm and are predominantly located within a region approximately 3 cm below the level of the vocal cords (Axtell and Mathisen, 2018). Excessive deposition of extracellular matrix (ECM) in ISGS leads to symptoms including cough and wheezing, and a significant stridor and dyspnoea at rest in later stages of the disease (Axtell and Mathisen, 2018). Due to significant recurrence rates of up to 50% within about 500 days following endoscopic treatment, repeated surgical interventions are often performed to prevent severe airway obstruction (Gelbard et al., 2016a). Surgical resection with removal of all affected scar tissue provides the best long-term results with recurrence rates below 5% (Tierney et al., 2023). Recent investigations into the pathomechanisms of ISGS revealed a high number of infiltrating T-cells accompanied by the activation of the IL-17A/IL23 pathway (Gelbard et al., 2016b). Further research uncovered a direct influence of IL-17A on fibroblast proliferation in ISGS, along with an interplay with TGF-ß in regulating collagen production by fibroblasts (Morrison et al., 2019). A potential association between a specific upper airway microbiota, especially members of the Moraxellaceae family, and ISGS has also been discussed (Hillel et al., 2019). In contrast to patients with granulomatosis with polyangiitis a human leucocyte antigen (HLA) association with allele DPB1*04:01 or allele homozygosity was excluded in ISGS (Rohlfing et al., 2023). Among ISGS patients, clonality of the TCR repertoire is driven by CD8^+^ T-cells, and ISGS patients possess numerous TCRs targeting viral and intercellular pathogens. High frequency clonotypes do not map to known targets in public datasets (Clark et al., 2023).

There is a notably high incidence observed in Caucasian female perimenopausal individuals (Gelbard et al., 2016a). Interestingly, premenopausal patients were shown to have a more aggressive disease variant than peri- and postmenopausal patients. However, it is unclear whether this is related to reduced estrogen in the peri- and postmenopausal state or the age-related physiology of wound healing and inflammation, regardless of estrogen (Nanda et al., 2024).

At the pathophysiological level, numerous questions regarding the diseases are still unanswered. Recent studies found parallels of ISGS and other fibrotic diseases (Marchioni et al., 2022; Sharif et al., 2023). The application of newly developed methods such as single cell RNA sequencing (scRNAseq) provides important insights into the prevailing cellular and transcriptional situation of multiple pathologies (Gelbard et al., 2023). Using this method, research groups have decoded the pathologic airway epithelium of ISGS and identified S100A8/A9 as a key biomarker of ISGS macrophages (Berges et al., 2023; Gelbard et al., 2023; Ospino et al., 2023). Fibrotic diseases are typically driven by multiple different cell types, with fibroblasts assuming a crucial role as the primary producers of the ECM in most cases (Henderson et al., 2020). Transcriptional studies already identified pro-fibrotic subtypes of fibroblast in distinct fibrotic diseases (Habermann et al., 2020; Deng et al., 2021; Vorstandlechner et al., 2021). Beyond the well-established role of fibroblasts, recent discoveries have highlighted the involvement of Schwann cells in fibrotic processes (Parfejevs et al., 2018; Direder et al., 2022a). These profibrotic Schwann cells exhibit a specific molecular pattern, contributing the matrix formation and influence other cell types, particularly macrophages (Direder et al., 2022a; Direder et al., 2022b).

In this study, we performed single cell RNA sequencing utilizing resected specimens of patients suffering from ISGS, to unravel the cellular and transcriptional composition of ISGS compared to healthy, unaffected tracheal tissue. We hypothesize that an unbiased analysis of the transcriptional datasets may yield novel insights into the pathology of ISGS, providing new information on potential pathologic cellular states and disease-induced changes of the prevailing cellular environment. Our study depicts the cellular composition of ISGS and healthy trachea and uncovers celltype specific transcriptional difference between the two conditions. The findings additionally confirm the presence of pathologic, pro-fibrotic fibroblasts in the ISGS and unveil their transcriptional pattern. The results of this study further suggest a potential involvement of Schwann cells in this fibrotic disease and provide information on a pathological increase of plasma cells in ISGS.

## 2 Materials and methods

### 2.1 Sample acquisition

Tissue samples of idiopathic subglottic stenosis and of unaffected, healthy tracheal areas were obtained from women who underwent open surgery with resection of the affected tracheal segment (donor information—Supplementary Table S1). A total of six patients was included, whereby paired samples of diseased and healthy tissues were obtained from five. Tissue of healthy trachea has been obtained including cartilage, whereas the tissue of the stenosis was pure fibrotic tissue. For further processing, the cartilage was removed as much as possible using a scalpel. Patients with previous chemotherapeutic or radiation treatment were excluded. Only surplus tissue not required for pathologic examination was used in this study. Written informed consent was obtained from all donors.

### 2.2 Cell isolation and generation of cell suspension

Upon tracheal resection, tissues were cooled and immediately processed. Samples were washed with sterile Dulbecco`s phosphate-buffered saline (PBS, without Ca^2+^ and Mg^2+^, Gibco, Thermo Fisher Scientific, Waltham, MA, United States), mechanically minced, and enzymatically dissociated using MACS Miltenyi Multi Tissue Dissociation Kit 1 (Miltenyi Biotec, Bergisch Gladbach, Germany) in accordance with the manufacturer’s instructions. Cell aggregates were dissociated using gentleMACS OctoDissociator (Miltenyi) with the standard gentleMACs program “37C_tdk_1.” Afterwards, cell suspensions were passed through 70 and 40 μm cell strainers and washed twice with 0.04% bovine serum albumin (BSA, Sigma Aldrich, St. Louis, MO, United States) in PBS. Cell number and viability were determined using a LUNA-FL™ Dual Fluorescence Cell Counter (Logos Biosystems, Anyang-si, Gyeonggi-do, South Korea) and the Acridine Orange/Propidium Iodide (AO/PI) Cell Viability Kit (Logos Biosystems). Only cell suspensions displaying a viability >80% were further processed and cell counts were set to 0.7–1.0 × 10^6^ cells/mL. The isolation process was performed for all samples within 4 h after resection and permanent cooling to minimize cell damage.

### 2.3 Single cell RNA sequencing

Single cell suspensions were further used for Gel Beads-in-emulsion (GEM) preparation, cDNA amplification and library preparation was performed using the Chromium Next GEM Single Cell 5′ Kit v2, the Dual Index Kit TT Set A (all 10x Genomics, Pleasanton, CA, United States), Chromium Next GEM Chips type K (10X Genomics) and the Chromium controller (10X Genomics), as described previously (Direder et al., 2022a). RNA sequencing, demultiplexing and counting was performed by the Biomedical Sequencing Core Facility of the Center for Molecular Medicine (CeMM Research Center for Molecular Medicine, Vienna, Austria). Samples were sequenced (read length 50 bp) on a NovaSeq 6000 (Illumina, San Diego, CA, United States). Raw reads were demultiplexed, aligned to the human reference genome (GrCh38-2020-A) and counted using the Cellranger pipeline (Cellranger v.6.1.2, 10x Genomics).

### 2.4 Bioinformatics analysis

For Bioinformatics analyses, R (R v.4.0.3, The R Foundation, Vienna, Austria), R-studio and Seurat (Seurat v.4.3.0.1, Satija wellLab) were used (Hao et al., 2021).First, all dataset features were proven for their annotation as NCBI symbol according to the *Homo sapiens* Ensemb ID and potential transcript duplicates were identified by their ident rowIDs and removed. Datasets were converted into Seurat objects. To remove apoptotic cells and erythrocytes from analyses, only cells displaying <5% mitochondrial counts and <5% hemoglobin subunit beta (*HBB*) counts were included in the study. In total, 5,430 cells from ISGS 1, 564 cells from ISGS 2, 3,773 cells from ISGS 3, 762 cells from HT 1 and 2,394 cells from HT two passed the quality control for further analyses. The purified data were pre-processed using sctransform-normalization with the glmGamPoi package and subsequently, all datasets were joined into a list and integrated using the commands using “SelectIntegrationFeatures” with nfeatures = 3,000, “PrepSCTIntegration,” “RunPCA,” “FindIntegrationAnchors” with “SCT” as normalization.method and “rpca” as reduction and “IntegrateData” with “SCT” as normalization method as recommended by the Seurat Vignette (Hafemeister and Satija, 2019; Ahlmann-Eltze and Huber, 2021). Next, PCA with npcs = 50 and UMAP with 1:30 dimensions were calculated. “FindNeighbors” was applied with 1:30 dimensions and “FindClusters” with a resolution of 0.1. The “FindAllMarkers” command with default setting was performed to identify cluster-specific genes and together with well-established marker genes the identified cluster were annotated (marker genes information—Supplementary Table S2). UMAPs were constructed using the command “UMAPPlot”, Dotplots by the command “DotPlot,” Violinplots by “VlnPlot” and heatmaps by the command “DoHeatmap.” The average expression depicted in the Dotplots are scaled data based on the normalized and integrated datasets. Differentially expressed gene analysis between ISGS and Healthy trachea were performed comparing cell type specific datasets of the same patients (ISGS 2 vs. HT 1; ISGS 3 vs. HT 2) and for the unmatched patient data were compared with both controls datasets (ISGS 1 vs. HT 1 and HT 2). Resulting lists were compared and only genes with p_val_adj < 0.05 and/or avg_log2FC > 0.58, present in at least two out of three lists were depicted in the designed graphic. GO-Term analyses were performed applying Enrichr and Metascape (https://metascape.org; accessed on 2023-12-21) (Chen et al., 2013; Kuleshov et al., 2016; Zhou et al., 2019; Xie et al., 2021). For Enrichr only identified genes with average fold change >2 were included. For Metascape analysis, a *p*-value cutoff of 0.05 and a minimum enrichment score of two were defined as significant. All subset analyses were performed based on the raw data of the selected cell cluster. Subset analyses were performed using the previously mentioned command sequence with 1:25 dimensions and a resolution of 0.2. Module scores to identify cell types included in extracellular matrix formation were composed including the whole gene list for each category published by Naba et al. (2016). Subtype characterization was conducted using published cell subtype-specific genes (Ascensión et al., 2021; Direder et al., 2022b). The annotation of fibroblast subtypes has been performed applying “AddModuleScore” composed by the distinct published gene signatures of Ascensión et al. (2021). The subtype annotation of plasma cells according to published subtype marker genes was not sufficient, therefore a consecutive numbering of the clusters was chosen. Potential cell-cell interactions were identified by CellChat performed according to the published vignette (Jin et al., 2021). For the command “filterCommunication” a min.cells of 35 was chosen. Mentioned *p*-values and communication probability resulted from default settings with a *p*-value threshold of 0.05.

### 2.5 Immunofluorescence and hematoxylin and eosin staining

In total three tissue samples of each condition were washed with PBS and fixed in 4.5% formaldehyde solution, neutral buffered (SAV Liquid Production GmbH, Flintsbach am Inn, Germany) for 24 h at 4°C, directly following surgery. On the next day, the samples were washed with PBS overnight and then dehydrated by step-wise incubation with 10%, 25%, and 42% sucrose solution, each overnight at 4°C. The samples were snap-frozen using optimal cutting temperature compound (OCT compound, TissueTek, Sakura, Alphen aan den Rijn, Netherlands) and preserved at −80°C. Sections of 10 µm were cut with a cryotome (Leica, Wetzlar, Germany), dried for 30 min and immersed in PBS. Permeabilization and blocking of the sections was performed for 15 min using 1% BSA, 5% goat serum (DAKO, Glostrup, Denmark) and 0.3% Triton-X (Sigma Aldrich) in PBS. Details for antibodies, dilutions and incubation times can be found in Supplementary Table S3. Antibodies not ready-to-use were diluted in antibody staining solution (1% BSA, 0.1% Triton-X in PBS). Incubation with secondary antibodies was performed for 1 h in combination with 50 μg/mL 4,6-diamidino-2-phenylindole (DAPI, Thermo Fisher Scientific). Next, sections were mounted with appropriate medium (Fluoromount-G, SouthernBiotech, Birmingham, AL, United States) and stored at 4°C. Imaging was performed with a confocal laser scanning microscope (TCS SP8X, Leica) equipped with a 20x (0.75 HC-Plan-Apochromat, Multimmersion), a 20x (0.75 HC- Plan-Apochromat) and a 63x (1.3 HC-Plan-Apochromat, Glycerol) objective using Leica application suite X version 1.8.1.13759 or LAS AF Lite software (both Leica) and an Olympus BX63 microscope (Olympus, Tokyo, Japan) with Olympus CellSens Dimension v2.3 (Olympus) software with standardized exposure time for all samples. A maximum projection of total z-stacks is depicted in the confocal images. Hematoxylin and eosin staining (H&E) of healthy trachea and ISGS tissue was performed according to standard protocol.

### 2.6 Fiber alignment examination

For fiber alignment analysis, H&E staining of three ISGS and three healthy trachea tissue slides were imaged and examined using Curvealign V4.0 Beta (MATLAB software, Cleve Moler, MathWorks, Natick, Massachusetts, United States). Fiber contrast, brightness and color were optimized by Adobe Photoshop CS6 (Adobe, Inc., San Jose, CA, United States). Three regions of interest (size 256 pixels, 256 pixels) were analyzed per image. For statistical evaluation, the coefficient of alignment was used.

### 2.7 Statistics

GraphPad Prism eight software (GraphPad Software Inc., La Jolla, CA, United States) was used for statistical evaluations. Student’s t-test was applied to compare two normally distributed groups. Fisher’s exact test was performed to compare relative cell numbers of distinct cell types. For potential cell-cell interactions, *p*-values were computed from one-sided permutation tests according to commands default settings. Wilcoxon test was applied to identify differentially expressed genes. *p*-values were marked in figures by asterisks. *p < 0.05, **p < 0.01, ***p < 0.001 and ****p < 0.0001.

## 3 Results

### 3.1 scRNAseq identifies the cellular composition of ISGS tissue

**FIGURE 1:**
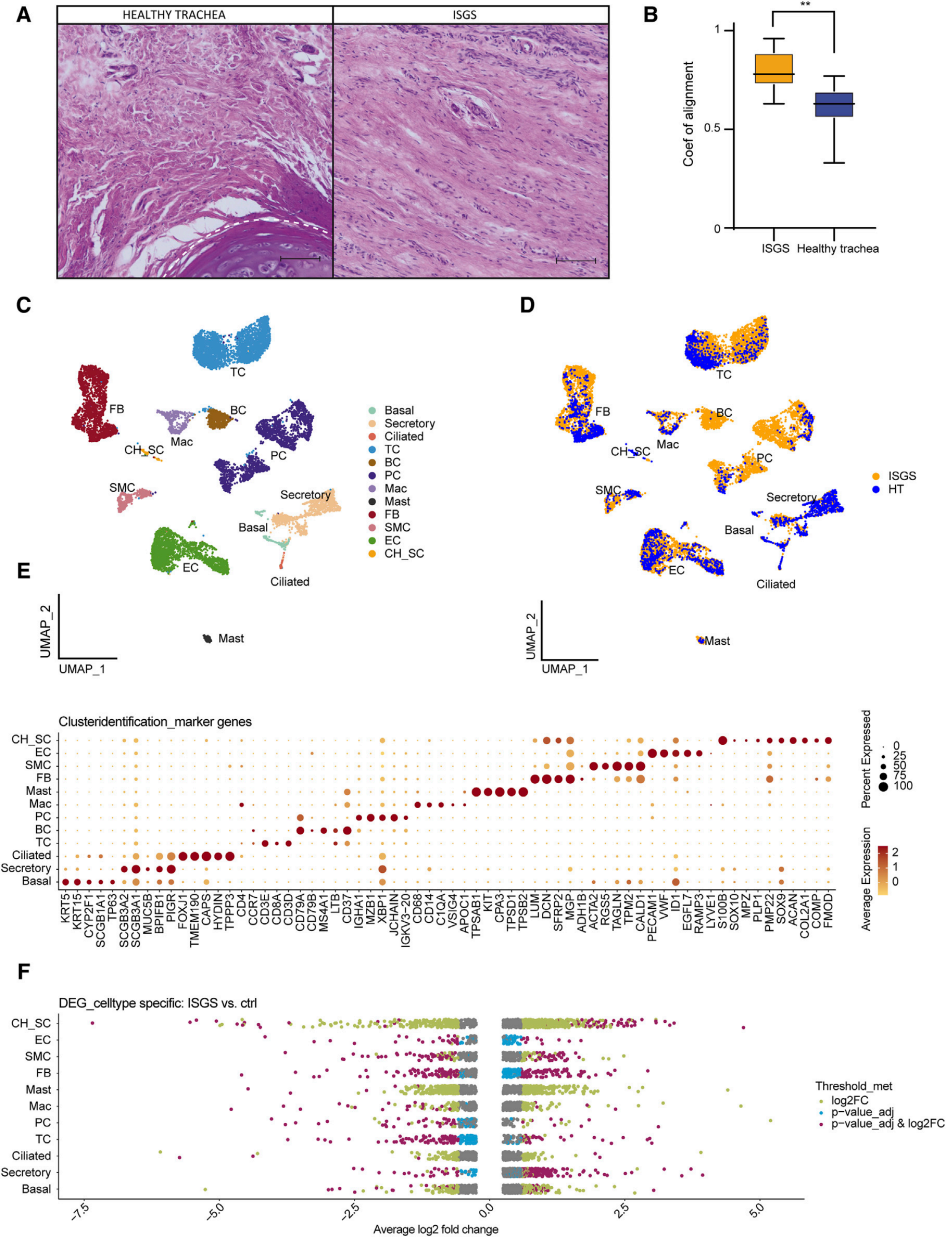
Single cell RNA sequencing of healthy trachea and ISGS. **(A)** Hematoxylin-eosin staining of healthy trachea and ISGS. Scale bars: 250 µm. Dashed line indicates soft tissue-cartilage border **(B)** Boxplot of the alignment coefficient evaluation of healthy trachea and ISGS. The middle line depicts the arithmetic mean. **p < 0.01 **(C)** UMAP-Plot after integration of all datasets. Clusters were characterized as basal cells (Basal), secretory cells (Secretory), ciliated cells (Ciliated), T-cells (TC), B-cells (BC), plasma cells (PC), macrophages (Mac), mast cells (Mast), fibroblasts (FB), smooth muscle cells (SMC), endothelial cells (EC), Chondrocyte-Schwann cells (CH_SC). **(D)** UMAP-Plot after integration of all datasets coloured by tissue. **(E)** Dotplot depicting expression of well-known marker genes for cluster characterization. **(F)** Strip-Plots showing differentially expressed genes of all different clusters comparing ISGS with healthy trachea. Gene expression thresholds are colour coded: adjusted *p*-values ≤0.05 and log2(FC) ≥0.58 (purple), adjusted *p*-values ≤0.05 only (blue), log2(FC) ≥0.58 only (green), not significant (grey). Cell cluster with low cell number, leading to no meaningful results, were excluded.

To elucidate the cellular composition of ISGS compared to the adjacent unaffected tissue and to identify previously uncharacterized cellular and transcriptional irregularities that may influence the progression of the fibrotic disease, scRNAseq was conducted. In line with previous publications, ISGS was characterized by areas with particularly high cellular infiltrations (Figure 1A) (Grillo et al., 1993). Additionally, an augmented ECM featuring enhanced fiber alignment was detectable in ISGS (Figures 1A, B). Through scRNAseq, we obtained data from a total of 12,723 cells from three ISGS and two healthy trachea samples for subsequent bioinformatics analyses (Supplementary Table S1). Following data pre-processing, 12 distinct cell clusters were identified each consisting of cells from both conditions (Figures 1C, D; Supplementary Figure S1). These cell clusters were characterized, using well-established cell markers in combination with their identified cluster marker, as basal cells (Basal), secretory cells (Secretory), ciliated cells (Ciliated), T-cells (TC), B-cells (BC), plasma cells (PC), macrophages (Mac), mast cells (Mast), fibroblasts (FB), smooth muscle cells (SMC), endothelial cells (EC) (Figure 1E; Supplementary Figure S2). One cluster expressed gene sets typical for chondrocytes and Schwann cells (CH_SC) (Figure 1E; Supplementary Figures S2–S4).

Analysis of differentially expressed genes revealed substantial differences between ISGS and the normal trachea. The most pronounced transcriptional differences were observed in FB (Figure 1F). Comprehensive lists of all identified differentially expressed genes (DEGs) are available in the supplements (Supplementary Table S5).

### 3.2 Plasma cells with high subtype variety accumulate in ISGS

**FIGURE 2:**
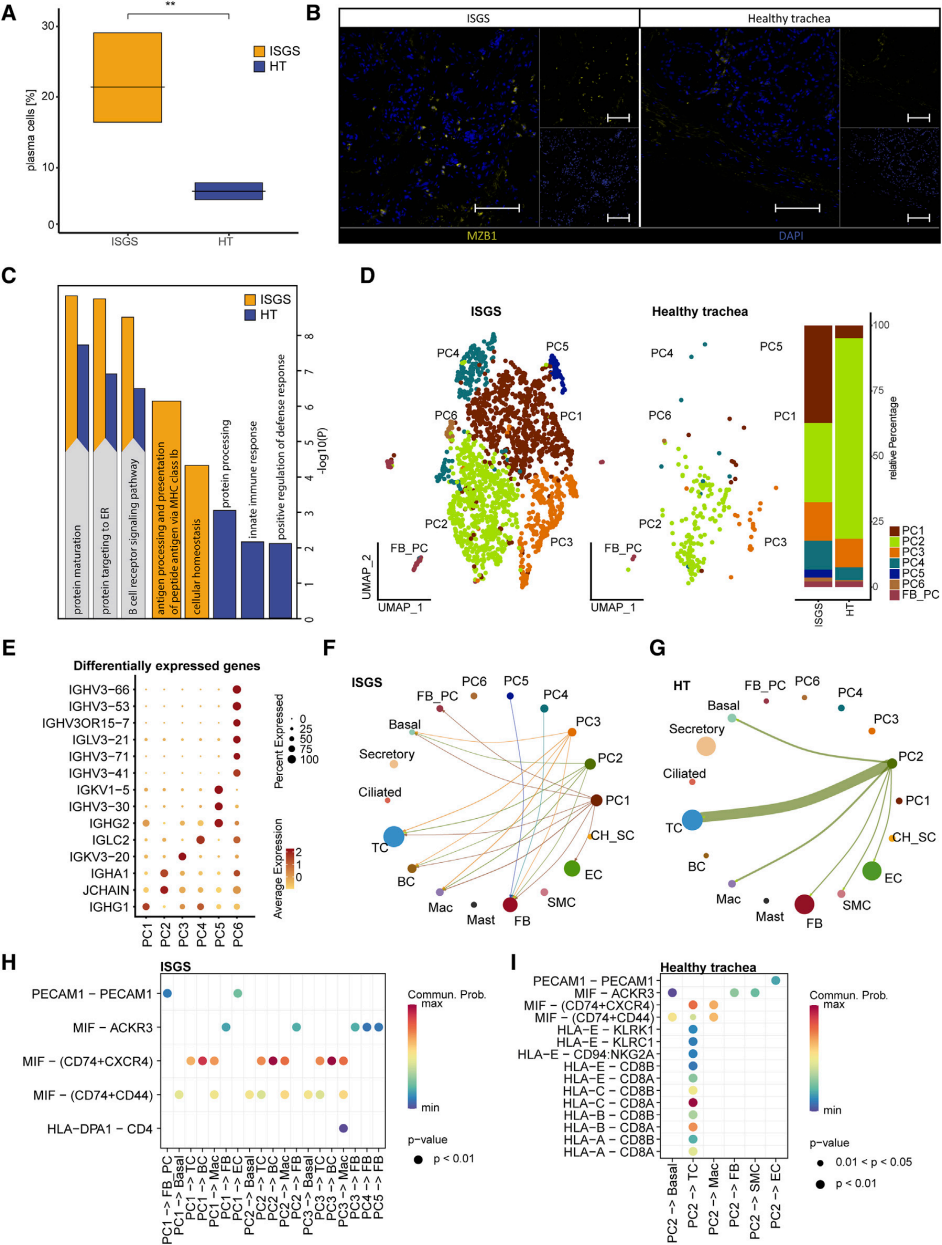
Transcriptional analysis reveals increased amount of plasma cells in ISGS tissue. **(A)** Boxplot depicting relative amount of plasma cells in the transcriptional datasets. **(B)** Representative immunofluorescence staining of Marginal zone B and B1 cell-specific protein (MZB1)-positive plasma cells in ISGS and healthy trachea, Tissues of n = 3 donors per condition were stained. Scale bars: 100 µm **(C)** GO-Term enrichment of differentially expressed genes with average foldchange ≥1.5 comparing plasma cells of ISGS and healthy trachea with secretory cells as neutral reverence cluster respectively. Bar-length depicts statistical significance of the term. Identical relevant terms identified in both analysis (left) and relevant individual terms (right), ISGS results are colored in yellow, healthy trachea in blue. **(D)** UMAP-Plot of the subset of PC reveals distinct PC-subcluster: PC1-PC6, Barplot indicates relative amounts of subcluster within ISGS and healthy trachea. **(E)** Dotplot shows differentially expressed genes with foldchange ≥2 of the distinct PC subcluster. **(F–G)** Circle plots of identified interactions from distinct PC-subtypes with the remaining cell types in ISGS and healthy trachea. Bow thickness represents amount of interactions **(H–I)** Dotplots depict the detected receptor-ligand couples of PC-subtypes with remaining cell types in ISGS and healthy trachea tissue.

A comparison of the relative cellular distribution within the different samples revealed a remarkable cellular presence of PCs in ISGS (18.7%, 16.1%, 29.4%), whereas in the healthy trachea, the presence of PCs was below 9% in each sample (Figure 2A). IF staining of the PCs, using MZB1 as marker revealed spots of increased PC presence in the ISGS tissue, whereas in unaffected tissue, the abundance of PC was scarce (Figure 2B). The utilization of GO term analysis, focusing on the top 30 genes expressed in PCs under both conditions, indicated a heightened engagement of cellular processes linked with protein maturation and B-cell receptor signaling pathway in PCs from ISGS (Figure 2C).

Subsetting of the PC cluster uncovered six transcriptionally distinguishable PC-subtypes (PC1-PC6) in ISGS (Figure 2D). A more precise annotation of the detected subclusters according to previously described PC subtypes was not feasible (Supplementary Figure S4) (Khodadadi et al., 2019; Rossi et al., 2021). PC2 appeared to constitute the predominant PC type in healthy tissue (Figure 2D). The cluster marker list indicated a distinct expression of genes crucial for immunoglobulin production, such as *IGHG1* in PC1, *IGHA1* in PC2, *IGKV3-20* in PC3 and *IGLC2* in PC4, among others (Figure 2E; Supplementary Figure S4). Analysis of potential cell-cell interactions showed a more diverse interaction of PCs in ISGS with other cell types than in the healthy tissue (Figures 2F, G). A high probability of a crosstalk between PCs and BCs via MIF-(CD74+CXCR4) was exclusively detected in ISGS, a receptor pathway involved in wound-healing and recovery of multiple organs (Figure 2H; Supplementary Figures S5A, B) (Farr et al., 2020). In the healthy trachea, only PC2 revealed recognizable cell-cell interactions, mainly typical immunological interactions with TCs (Figure 2I; Supplementary Figures S5C, D). Our results indicate a highly heterogeneous population of PCs in ISGS. Their different expression patterns, especially of genes important for immunoglobulin production, suggest an involvement of specific immunoglobulins in the pathogenesis of ISGS.

### 3.3 Fibrotic fibroblasts affect tissue environment in ISGS

**FIGURE 3:**
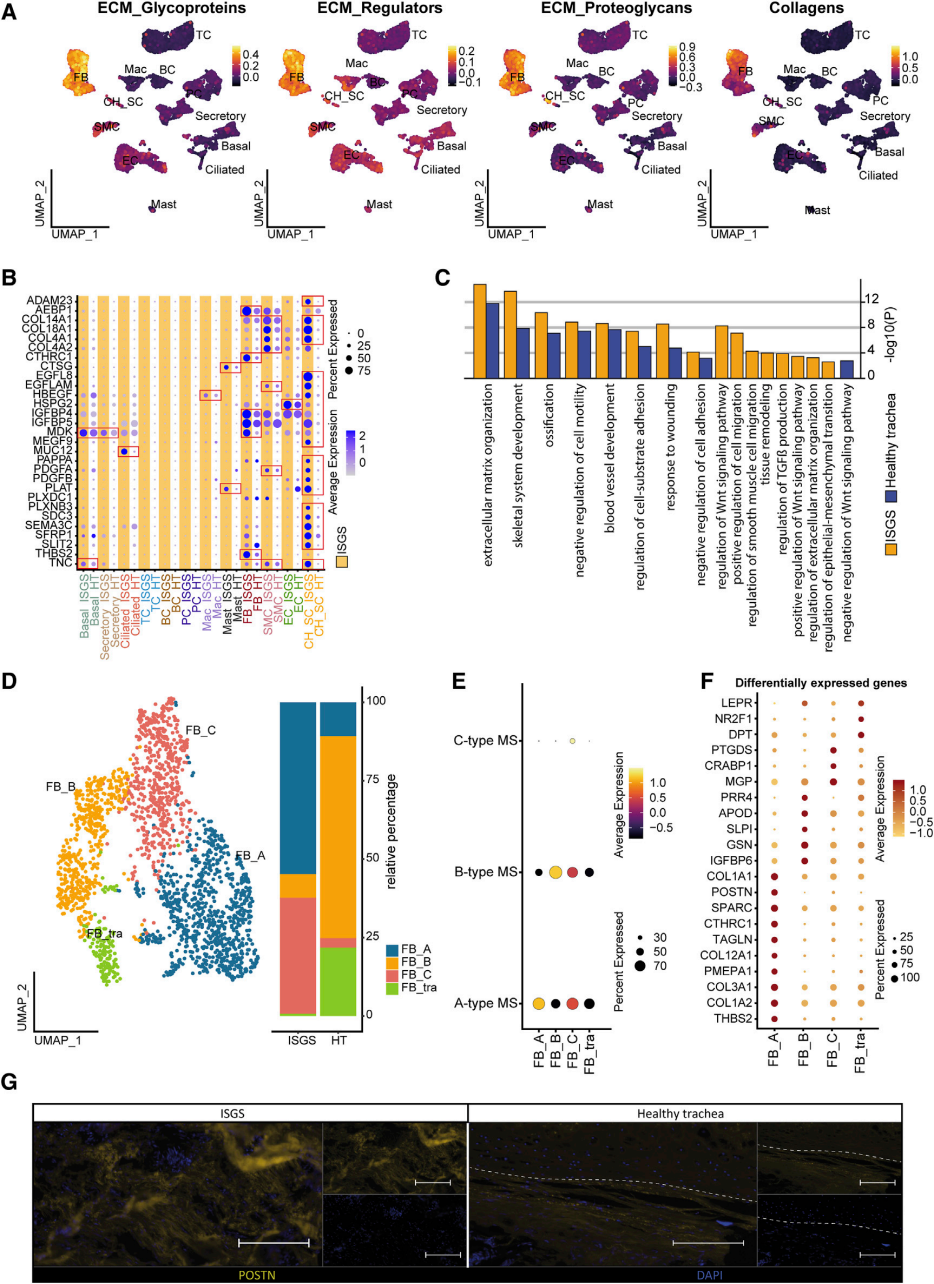
Impact of Fibroblasts on ISGS pathology. **(A)** Feature Plots showing module scores of extracellular matrix (ECM) -associated gene groups. **(B)** Dotplot depicting expression of matrix-associated genes by distinct cell types of healthy trachea and ISGS. **(C)** GO-Term enrichment of differentially expressed genes with average foldchange ≥2 comparing fibroblasts of ISGS with healthy controls. Identical relevant terms identified in both analysis (left) and relevant individual terms (right), ISGS results are colored in yellow, healthy trachea in blue. **(D)** UMAP-Plot of FB subclusters: FB-type A (FB_A), FB-type B (FB_B) and FB-type C (FB_C), tracheal specific FB subset (FB_tra). **(E)** Dot-Plot depicting module scores of published FB-subtype gene sets. **(F)** Dotplot shows differentially expressed genes with foldchange ≥2 of each FB subcluster. **(G)** Representative immunofluorescence image of periostin (POSTN) in ISGS and healthy trachea. Tissues of *n* = 3 donors per condition were stained. Dashed line indicates soft tissue-cartilage border, Scale bars: 100 µm.

To identify the cell types contributing most significantly to ECM production, we conducted module score analysis based on the recently published matrisome (Naba et al., 2016). This analysis revealed a substantial contribution of FB to ECM production, with potential matrix associated involvement of other cell clusters such as CH_SC (Figure 3A). A screening of ECM-associated genes unveiled multiple factors exhibiting upregulated expression in ISGS cells, including *CTHRC1*, *MUC12*, *PLAT*, *SFRP1*, *TNC* (Figure 3B; Supplementary Figure S6). Notably, the most pronounced differences in ECM gene expression were uncovered in the SC_CH cluster. Consequently, our data provide a transcriptional snapshot of the Figure 3B; Supplementary Figure S6 cellular landscape in ISGS, highlighting substantial transcriptional differences in matrix-associated gene expression.

Fibroblasts are recognized as a pivotal factor in fibrotic diseases, being the primary producer of ECM and influencing the function of other cell-types (Henderson et al., 2020). Given our data indicating significant alterations in gene expression associated with ECM formation in FBs in ISGS compared to the adjacent healthy tissue, we performed a detailed analysis of the FB cell cluster. To gain further insights into the functional role of the identified FBs, we conducted an enrichment analysis, revealing an enhanced involvement of ISGS-FBs in fibrotic features such as ECM organization, response to wounding and regulation of TGFß production (Figure 3C). Examination of FB cell-cell communication indicated only a slightly increased number of ligand-receptor interactions in ISGS compared to healthy trachea. However, notable differences were observed in the specific interactions identified (Supplementary Figure S7). Analyzing all resulting interactions indicated a high probability of communication between FBs, Mast and BCs. Notably, *COL4A2*, *MDK*, *NEGR1*, *POSTN*, *TNC*, *VEGFA* and *WNT5A* exclusively appeared in the FB interactions of the ISGS as communication partners. Subsetting of the identified FB cluster revealed several subclusters, which were classified according to the FB subtype scheme published by Ascension et al. (Ascensión et al., 2021) (Figures 3D, E). Cluster annotation revealed the presence of Type A (FB_A), Type B (FB_B) and Type C (FB_C) FBs in the samples. However, one FB cluster could not be explicitly assigned according to the Ascension nomenclature and was therefore designated as a tracheal specific FB subset (FB_tra) (Figures 3D, E). Comparing the cellular distribution in ISGS and healthy trachea, a pronounced prevalence of FB_A and FB_C was evident in the ISGS samples, whereas FB_B and FB_tra were predominant present in normal tissue (Figure 3D). Subsequent analysis revealed a strong expression of profibrotic genes, including *COL1A2*, *COL3A1*, *TAGLN*, *POSTN* and *COL1A1*, especially in the FB_A subset (Figure 3F). Immunofluorescence staining of periostin corroborated the increase of this pro-fibrotic factor in ISGS at the protein level (Figure 3G). These findings demonstrate the increased presence and the transcriptional profile of fibrotic FBs in ISGS.

### 3.4 In-depth analysis of Schwann cells suggests a pro-fibrotic impact on ISGS pathology

**FIGURE 4:**
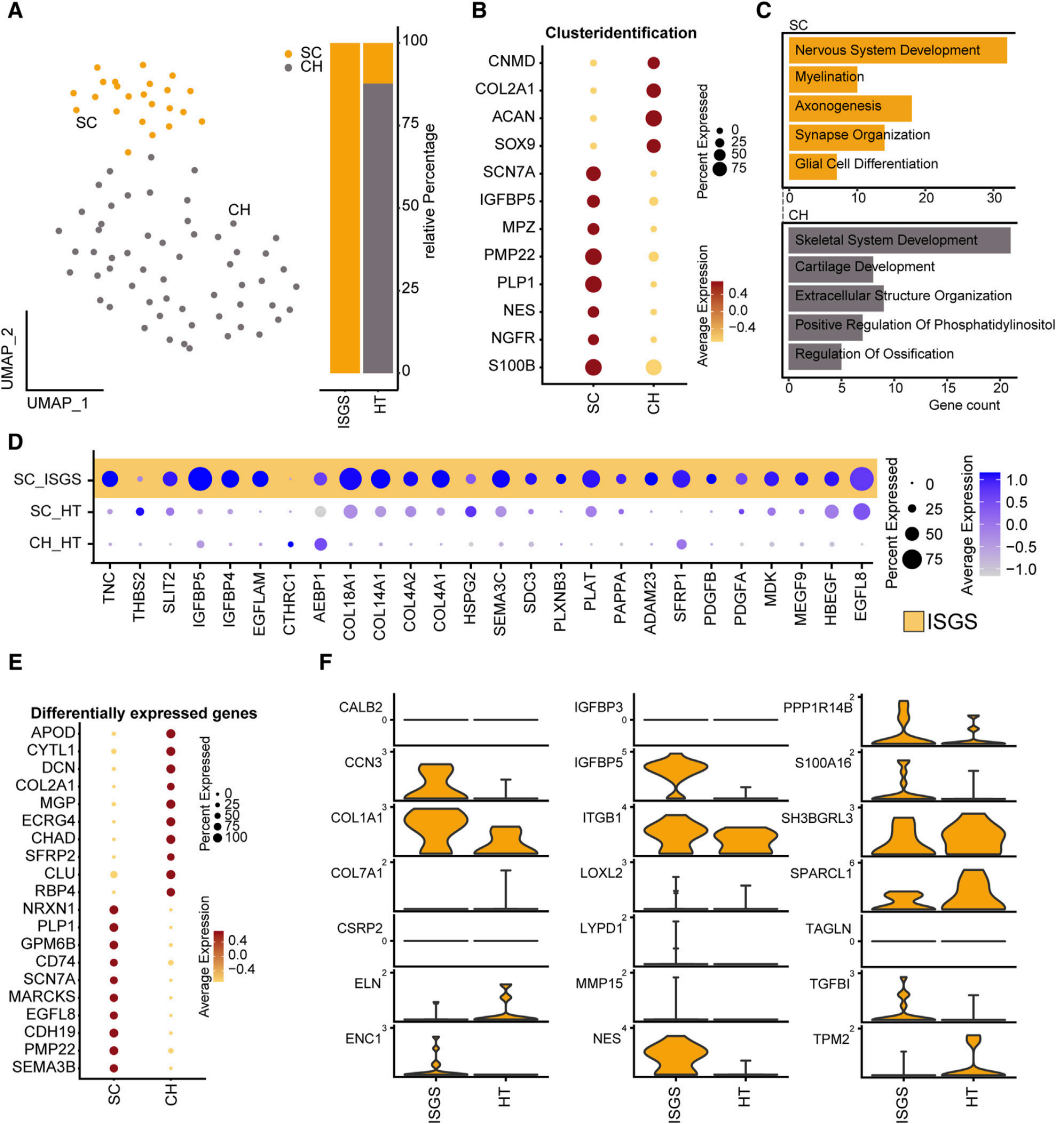
Transcriptional analysis reveals pro-fibrotic Schwann cells in ISGS. **(A)** UMAP-Plot of SC_CH-cluster. **(B)** Dotplot depicting expression of well-known marker genes for SC and CH. **(C)** Enrichment analysis of subset clustermarker with average foldchange ≥2. SC results depicted in yellow, CH in grey. **(D)** Dotplot shows differentially expressed genes comparing SCs of both conditions. **(E)** Dotplot of subset clustermarker genes with average foldchange ≥2. **(F)** Expression of pro-fibrotic SC-associated genes. Vertical lines depict maximum expression. Violin-width shows frequency of cells at the respective expression level.

A recent study of our group demonstrated a significant involvement of Schwann cell to fibrotic processes in pathologic cutaneous scars (Direder et al., 2022a). Therefore, we also conducted a detailed exploration of the SC_CH cluster with its distinctive expression pattern in our ISGS samples. Subset analysis of the SC_CH facilitated the segregation of the two cell types (Figure 4A). SCs were detected in both conditions, whereas CHs were only found in the unaffected tissue, as all fibrotic samples were obtained without any adherent cartilage (Figures 4A, B). Enrichr analysis of the two distinct clusters based on their cluster markers revealed cell type-specific features such as nervous system development and myelination for SCs and cartilage development and skeletal system development for CHs, supporting the annotation (Figure 4C).

**FIGURE 5:**
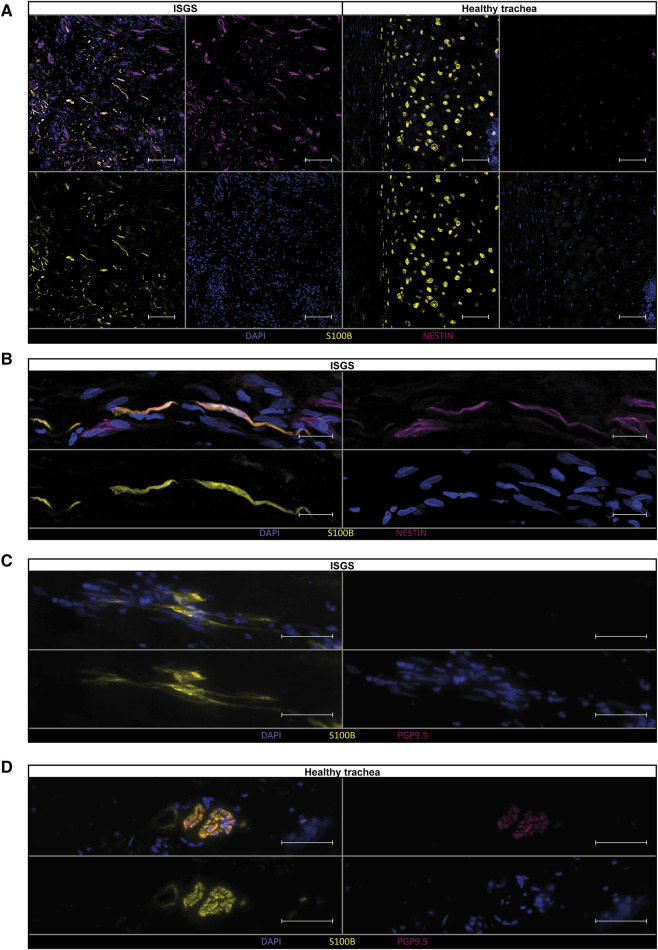
Verification of pro-fibrotic Schwann cells in ISGS tissue. Immunostainings of Schwann cells for **(A, B)** S100B and Nestin and **(C, D)** S100B and PGP9.5 shows double positive Schwann cells in ISGS compared to healthy trachea. In tissue of healthy trachea Schwann cells were only identified in nerve bundles **(D)**. One representative micrograph of *n* = 3 donors per condition is depicted. Scale bars in **(A)**: 100 µm, Scale bars in **(B–D)**: 50 µm.

Examination of the previously identified ECM-associated genes revealed a strong expression of these genes in SC of the ISGS, while SCs in healthy tissue and the detected CHs exhibit comparatively low expression levels (Figure 4D). The complete expression list of ECM-associated genes within the segregated SC_CH cluster is provided in the supplements (Supplementary Figures S8, S9). Transcriptional comparison of the two clusters showed a robust expression of characteristic SC genes (*PLP1*, *PMP22*, *SCN7A*) and CH genes (*DCN*, *COL2A1*). Interestingly, we also detected genes such as *EGFL8* and *CDH19*, which are known marker genes of SC precursors and repair SCs (Figure 4E) (Mirsky et al., 2008; Weiss et al., 2021). Recently, we identified the transcriptional pattern of profibrotic Schwann cells in pathologic scars (Direder et al., 2022b). Screening the detected SCs in ISGS and healthy tissue for these marker genes revealed the upregulation of nine out of 21 genes in SCs of the ISGS (Figure 4F). Remarkably, among these nine genes *IGFBP5*, *CCN3* and *NES* were included, three well-described main factors in profibrotic SCs. IF staining of SCs using S100B in combination with nestin uncovered double positive SCs in ISGS with a characteristic elongated form, comparable to those previously shown to be specific for pro-fibrotic SCs (Figures 5A, B) (Direder et al., 2022a). In healthy tissue, S100 staining was only observed in chondrocytes and large nerve bundles (Figures 5A, D), while nestin staining was negative. In addition, IF-staining of S100 in combination with PGP9.5, an established axon marker, revealed that most SCs present in ISGS were not associated with axons (Figure 5C), while all SCs present in healthy tracheas were associated with axons in nerve bundles. (Figure 5D). These findings collectively suggest the presence of dedifferentiated, activated SCs with a profibrotic function in ISGS.

## 4 Discussion

ISGS, characterized as a rare form of fibrotic disease, continues to elicit questions about its pathobiology. Specifically, the cellular drivers of this condition remain elusive. In this study, we employed sc RNAseq to elucidate the cellular composition of ISGS tissue, and compared it to normal, unaffected tracheal tissue. We aimed to identify cellular abnormalities and dysregulated mRNA expression that could potentially contribute to the disease pathology. On a histopathologic level, the disease is characterized by a dense fibrosis primarily constituted by concentrated cellular regions (Grillo et al., 1993). The histologic examination of our samples was consistent with this characterization.

Our scRNAseq analysis revealed a substantial higher abundance of PCs in ISGS. These cells were observed in specific areas as dense cellular spots. The identified PCs expressed *IGHG1* and *IGHG2*, which encode crucial proteins of Immunoglobulin (Ig)G (Bashirova et al., 2021). Plasma cells have already been recognized for their pivotal crucial role in a specific fibro-inflammatory disorder, a IgG4-related disease (Deshpande et al., 2012). In this pathology, PCs often exhibit significant positivity for IgG4, and fibrotic manifestations can occur in nearly any organ (Maritati et al., 2020). The presence of *IGHG1* and *IGHG2*-expressing PCs in ISGS suggests a disease-specific population of PCs, which differ from PCs commonly found in other fibrotic processes. The precise nature of these PCs and their contribution to ISGS requires further investigation in future studies.

In addition to IgG4-related fibrotic disease, an elevation of other IgG subclasses has also been reported in idiopathic pulmonary fibrosis, another fibrotic disorder in the thoracic region with an unsolved pathomechanism (Reynolds et al., 1977). In these cases, IgG accumulation is predominantly described as localized rather than systemic (Reynolds, 1988). Analysis of the broncho-alveolar lavage fluid from patients with idiopathic pulmonary fibrosis indicates a higher concentration of IgG1 and IgG3 compared to the healthy state (Reynolds, 1988). In addition, Prêle et al. recently reported the accumulation of PCs in fibrotic lungs, and depletion of PCs by the proteasome inhibitor bortezomib resulted in a reduced level of pulmonary fibrosis (Prêle et al., 2022). However, the mechanism leading to PC accumulation in fibrotic tissue and their precise function in pulmonary fibrosis still necessitate further research (Goodwin et al., 2022). These findings further support the previously postulated similarities between ISGS and pulmonary fibrosis (Sharif et al., 2023) and suggest potential therapeutic targets for interventions that affect this pathologic cell type in the pulmonary fibrosis also in the context of ISGS. The transcriptional pattern of PCs in ISGS did not reveal specific direct involvement in fibrotic processes; however, GO-term analysis unveiled hyper-activated basal function of the PCs. Moreover, potential intercellular connections indicated a robust interaction between macrophage migration inhibitory factor (MIF)-positive cells and those expressing CD74 and CXCR4. This interaction with B-cells involves a receptor-ligand combination previously reported to be implicated in fibrotic processes such as wound healing. It is tempting to speculate that interventions affecting this interaction could represent an alternative treatment option for ISGS in the future (Farr et al., 2020).

Our scRNAseq analysis also demonstrated that while FB cell numbers did not exhibit a significantly increase, their transcriptional profile exhibited the highest deviation from the healthy condition. Our investigation contributes to a deeper understanding of the subsets of FBs present in ISGS and their role in the disease. A-type FBs present in ISGS have been recognized for their involvement in ECM homeostasis, and dysregulation of this cell type is thought to contribute to fibrotic pathologies (Ascensión et al., 2021). In our analyses, it is conceivable that the normal, tracheal FBs (FB_tra) are represented in the healthy control samples, while FB_A, characterized by a significant upregulation of ECM genes such as *COL1A1*, *COL3A1*, *POSTN*, and others, appears to be in the pro-fibrotic more pathologic-like state. Consistent with our findings, Tsukui et al. recently described this specific set of genes to be upregulated in a pathologic FB cell cluster associated with pulmonary fibrosis (Tsukui et al., 2020). Collectively, our data suggest that specific FB subsets contribute to ISGS in a manner that parallels the common mechanisms observed in numerous other fibrotic organs. These data contribute to a deeper understanding of the subsets of FBs present in ISGS and their putative role to the disease.

Most notably, we identified a sub-population of SCs in ISGS exhibiting a pro-fibrotic phenotype. The pro-fibrotic role of SCs has recently been elucidated in keloids, a dermatological condition sharing hitherto unknown pathologic features (Direder et al., 2022a). In keloids, SCs exist in an activated state, promoting synthesis of extracellular matrix (Direder et al., 2022a). In our study, we detected the presence of SCs in ISGS with a specific gene expression pattern indicative of cell activation, featuring genes such as *IGFBP5* and *CCN3*, which are characteristic for pro-fibrotic SCs (Direder et al., 2022b). Additionally, these SCs expressed *EGFL8*, a gene secreted by repair-related SCs, implicated in neurite growth and neuronal differentiation (Tsukui et al., 2020). Another upregulated factor was *CDH19*, a gene known to be elevated in SC precursors during SC development, subsequently downregulated in the inactive state of SCs, whether myelinating or non-myelinating (Bashirova et al., 2021). On a histologic level, we identified cells exhibiting a morphology similar to pro-fibrotic SCs found in keloids (Direder et al., 2022a). Immunofluorescence staining indicated the presence of axon-independent SCs in ISGS. While the occurrence of SCs without contact to axons may not be as prominent as in keloids, our findings support the hypothesis of the involvement of axon free, pro-fibrotic SCs in diverse fibrotic diseases, including ISGS.

Fibrotic diseases like ISGS exhibit complex and unresolved pathomechanisms, involving interactions among multiple cell types in a pro-fibrotic state. As a result, establishing a simple pathological mechanism solely based on identified cellular observations is challenging, necessitating further extensive research. Our study, through the identification of plasma cells, fibroblasts, and Schwann cells, suggests potential parallels with other fibrotic diseases such as pulmonary fibrosis and keloids, indicating potential similarities in pathophysiological mechanisms. However, further investigations are needed to elucidate the interplay among these cell types and other cellular components.

The study encounters several limitations. Firstly, caution is required in analyzing three datasets of ISGS compared to two datasets from healthy tracheas using scRNAseq. Consequently, bioinformatics analyses comparing donors are included to strengthen the interpretation and significance of the results. While confirmation of the scRNAseq findings was performed through immunostaining experiments on a larger donor pool, it is important to note that this limitation was only partially addressed. Additionally, the restricted number of identified Schwann cells poses a challenge, given the demanding nature of Schwann cell isolation requiring lengthy enzymatic digestion (Weiss et al., 2018). Consequently, isolating more Schwann cells to increase cell numbers would likely impact other cell types significantly. Future studies focusing on Schwann cells in ISGS should meticulously evaluate optimal isolation methods to ensure the reliability of data obtained.

In addition, our study focuses on cell types directly involved in the fibrotic processes of ISGS, such as FBs and SCs, and those increased in number in ISGS, such as PCs. Consequently, the roles and subtype compositions of other cell types, as well as their potential involvement in non-fibrotic pathology-specific functions, remain to be elucidated in future studies.

In summary, our study elucidates the cellular and the transcriptional landscape of ISGS. It delineates the FB subsets involved in this fibrotic disease and identifies the pathologic FB-subtype present in the tissue based on its transcriptional pattern. Apart from FBs, PCs and SCs also appear to play an important role in the pathological processes of ISGS, existing in an activated state and potentially contributing to fibrosis. These findings may aid in the development of novel therapeutic strategies to treat ISGS.

## Data Availability

The data presented in the study are deposited in the NCBI’s Gene Expression Omnibus 435 (GEO) database (GEP series accession number GSE248105). [https://www.ncbi.nlm.nih.gov/geo/].
