# Supplementary material for: Transcriptional profiling sheds light on the fibrotic aspects of idiopathic subglottic tracheal stenosis
Source: Front Cell Dev Biol. 2024 Jul 12;12:1380902. doi: 10.3389/fcell.2024.1380902 (PMC11272577; doi:10.3389/fcell.2024.1380902)
Supplement: Supplementary file 1 [file Table1.PDF]

Supplementary Table 1 – donor information

ID	sample	Age	Sex	Method
ISGS 1	ISGS	83	Female	scRNAseq/Histo
ISGS 2 / HT 1	ISGS + healthy trachea	53	Female	scRNAseq/Histo
ISGS 3 / HT 2	ISGS + healthy trachea	46	Female	scRNAseq/Histo
ISGS 4 / HT 3	ISGS + healthy trachea	55	Female	Histo
ISGS 5 / HT 4	ISGS + healthy trachea	31	Female	Histo
ISGS 6 / HT 5	ISGS + healthy trachea	53	Female	Histo

Supplementary Table 1. donor information.
