# Supplementary material for: Transcriptional profiling sheds light on the fibrotic aspects of idiopathic subglottic tracheal stenosis
Source: Front Cell Dev Biol. 2024 Jul 12;12:1380902. doi: 10.3389/fcell.2024.1380902 (PMC11272577; doi:10.3389/fcell.2024.1380902)
Supplement: Supplementary file 2 [file Table2.PDF]

Supplementary Table 2 - marker genes information

celltype	abbreviation	marker gene	reference
basal cells	Basal	KRT5, KRT15, SCGB1A1, CYP2F1, TP63	(1)
secretory cells	Secretory	MUC5B, SCGB3A1, SCGB3A2, BPIFB1, PIGR	(2)
ciliated cells	Ciliated	FOXJ1, TMEM190, CAPS, HYDIN, TPPP3	(1, 2)
T-cells	TC	CD3E, CD8A, CD4, CCR7, CD3D	(3)
B-cells	BC	CD79A, CD79B, MS4A1, LTB, CD37	(2-4)
plasma cells	PC	IGHA1, MZB1, XBP1, JCHAIN, IGKV3-20	(2, 3)
macrophages	Mac	CD68, CD14, C1QA, VSIG4, APOC1	(2, 3)
Mast cells	Mast	TPSAB1, KIT, CPA3, TPSD1, TPSB2	(2, 3)
fibroblasts	FB	LUM, DCN, SFRP2, MGP, ADH1B	(2, 5)
smooth muscle cells	SMC	ACTA2, RGS5, TAGLN, TPM2, CALD1	(2, 5)
endothelial cells	EC	PECAM1, VWF, ID1, EGFL7, RAMP3, LYVE1	(2, 5)
Schwann cells	SC	S100B, SOX10, MPZ, PLP1, PMP22	(5)
chondrocytes	CH	SOX9, ACAN, COL2A1, COMP, FMOD	(6)

Supplementary Table 2. marker genes information.

1. Zuo WL, Rostami MR, Shenoy SA, LeBlanc MG, Salit J, Strulovici-Barel Y, et al. Cell-specific expression of lung disease risk-related genes in the human small airway epithelium. Respir Res. 2020;21(1):200.
2. Habermann AC, Gutierrez AJ, Bui LT, Yahn SL, Winters NI, Calvi CL, et al. Single-cell RNA sequencing reveals profibrotic roles of distinct epithelial and mesenchymal lineages in pulmonary fibrosis. Sci Adv. 2020;6(28):eaba1972.
3. Peng L, Jin X, Li BY, Zeng X, Liao BH, Jin T, et al. Integrating single-cell RNA sequencing with spatial transcriptomics reveals immune landscape for interstitial cystitis. Signal Transduct Target Ther. 2022;7(1):161.
4. Stewart A, Ng JC, Wallis G, Tsioligka V, Fraternali F, Dunn-Walters DK. Single-Cell Transcriptomic Analyses Define Distinct Peripheral B Cell Subsets and Discrete Development Pathways. Front Immunol. 2021;12:602539.
5. Direder M, Weiss T, Copic D, Vorstandlechner V, Laggner M, Pfisterer K, et al. Schwann cells contribute to keloid formation. Matrix Biol. 2022.
6. Gan Y, He J, Zhu J, Xu Z, Wang Z, Yan J, et al. Spatially defined single-cell transcriptional profiling characterizes diverse chondrocyte subtypes and nucleus pulposus progenitors in human intervertebral discs. Bone Res. 2021;9(1):37.
