# Supplementary material for: Transcriptional profiling sheds light on the fibrotic aspects of idiopathic subglottic tracheal stenosis
Source: Front Cell Dev Biol. 2024 Jul 12;12:1380902. doi: 10.3389/fcell.2024.1380902 (PMC11272577; doi:10.3389/fcell.2024.1380902)
Supplement: Supplementary file 3 [file Table3.PDF]

Supplementary Table 3 – antibody information

1° Antibodies					
Antigen	Species	catalog No	company	dilution	incubation
S100	rabbit	#Z0311	DAKO	ready to use	o.n., 4°C
Nestin	mouse	#MAB5326	Millipore	1:200	o.n., 4°C
PGP9.5	mouse	#7863-1004	BioRad	1:250	o.n., 4°C
POSTN	rabbit	#EPR19934	abcam	1:2000	o.n., 4°C
MZB1	rabbit	#HPA052694	merck	1:100	o.n., 4°C
**2° Antibodies**					
Antigen	Species	catalog No	company	dilution	incubation
α rb AF488	goat	#A32731	Sigma - Aldrich	1:600	1 hr, RT
α m AF546	goat	#A21123	Thermo Fisher	1:400	1 hr, RT
α g AF546	donkey	#A11056	Sigma – Aldrich	1:400	1 hr, RT
