# Supplementary figures and images for: Transcriptional profiling sheds light on the fibrotic aspects of idiopathic subglottic tracheal stenosis
Source: Front Cell Dev Biol. 2024 Jul 12;12:1380902. doi: 10.3389/fcell.2024.1380902 (PMC11272577; doi:10.3389/fcell.2024.1380902)

Figure S1

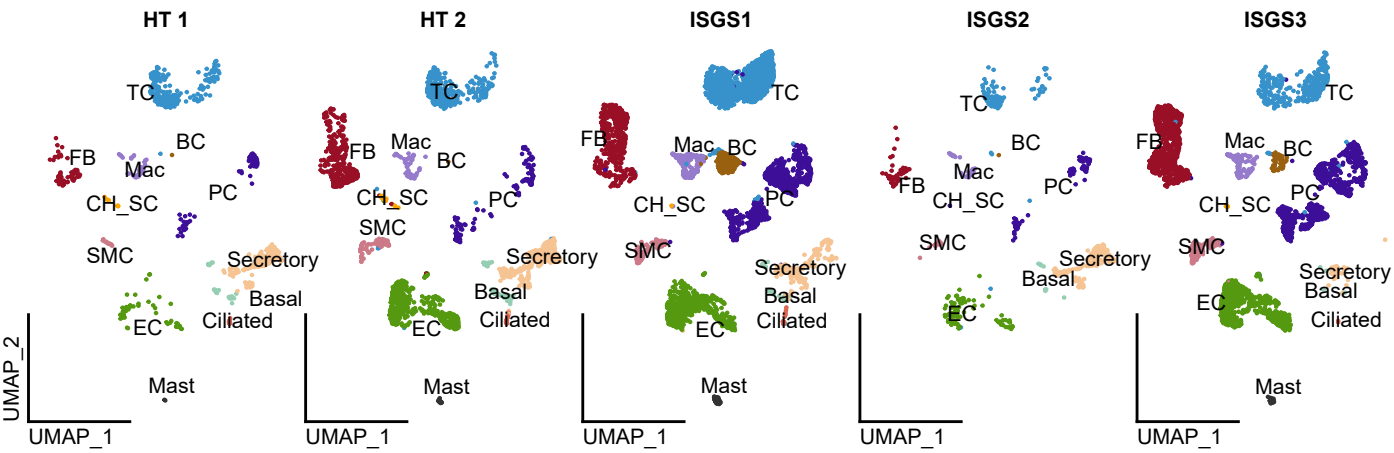

Figure S2

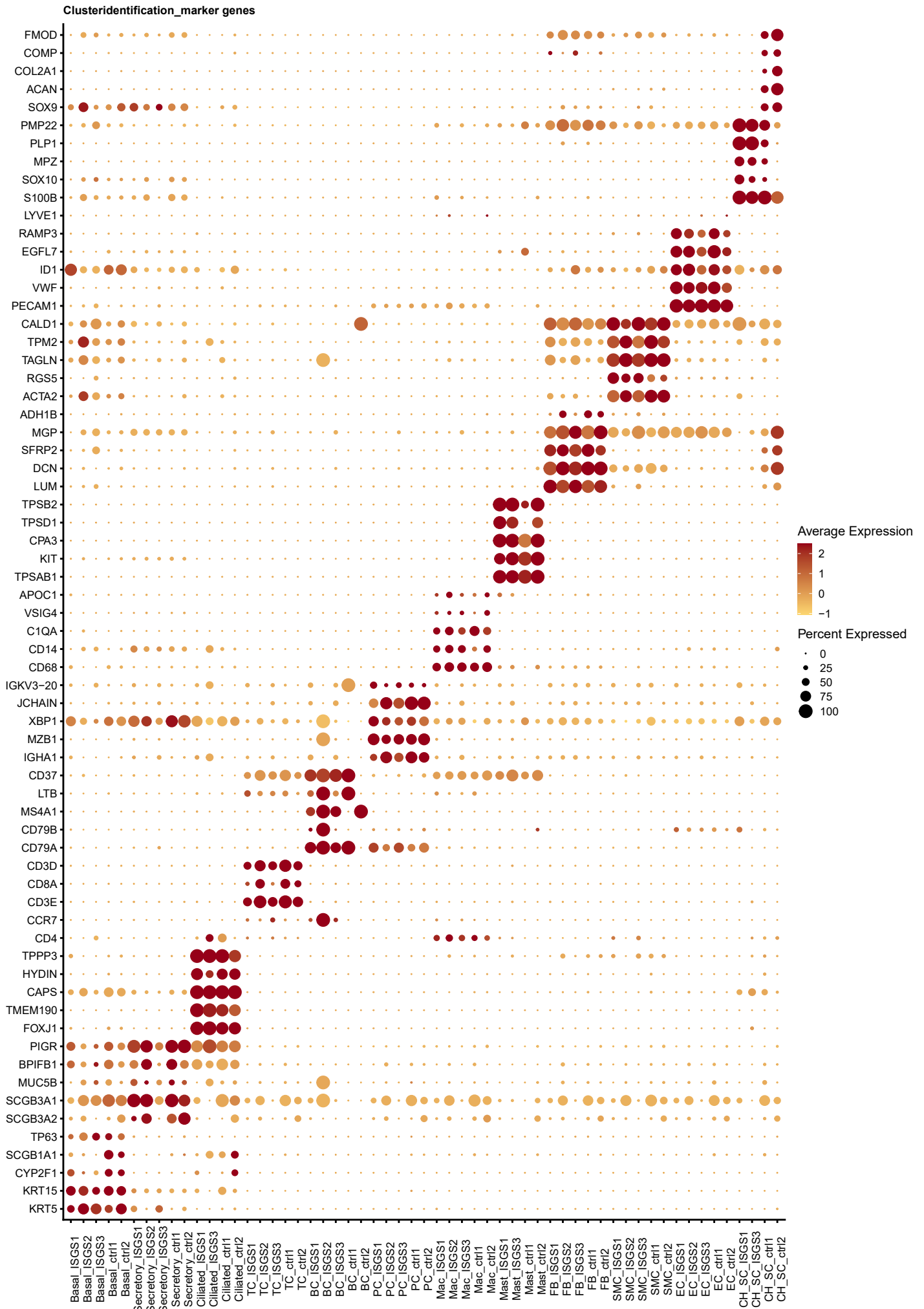

Figure S3

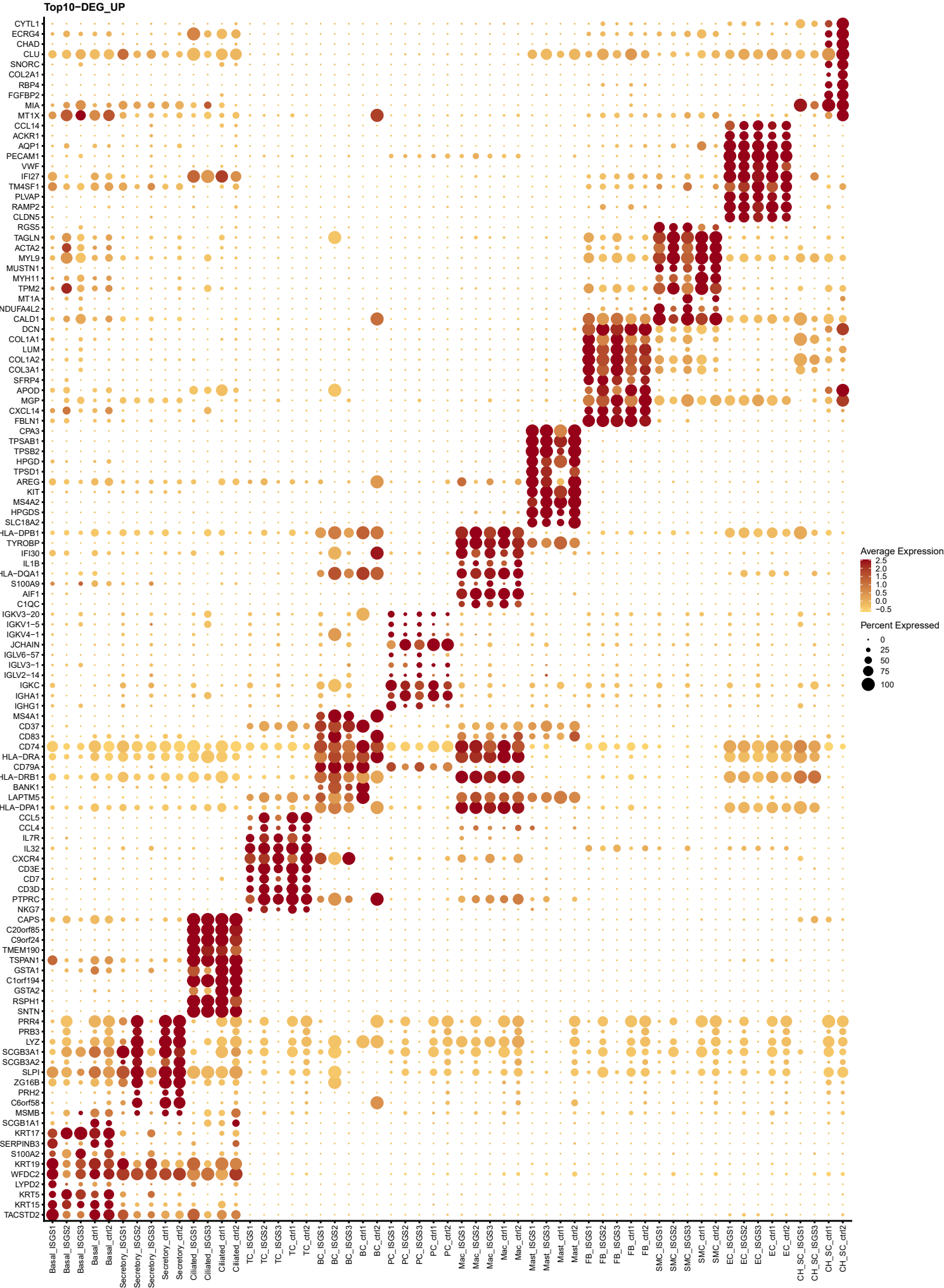

Figure S4

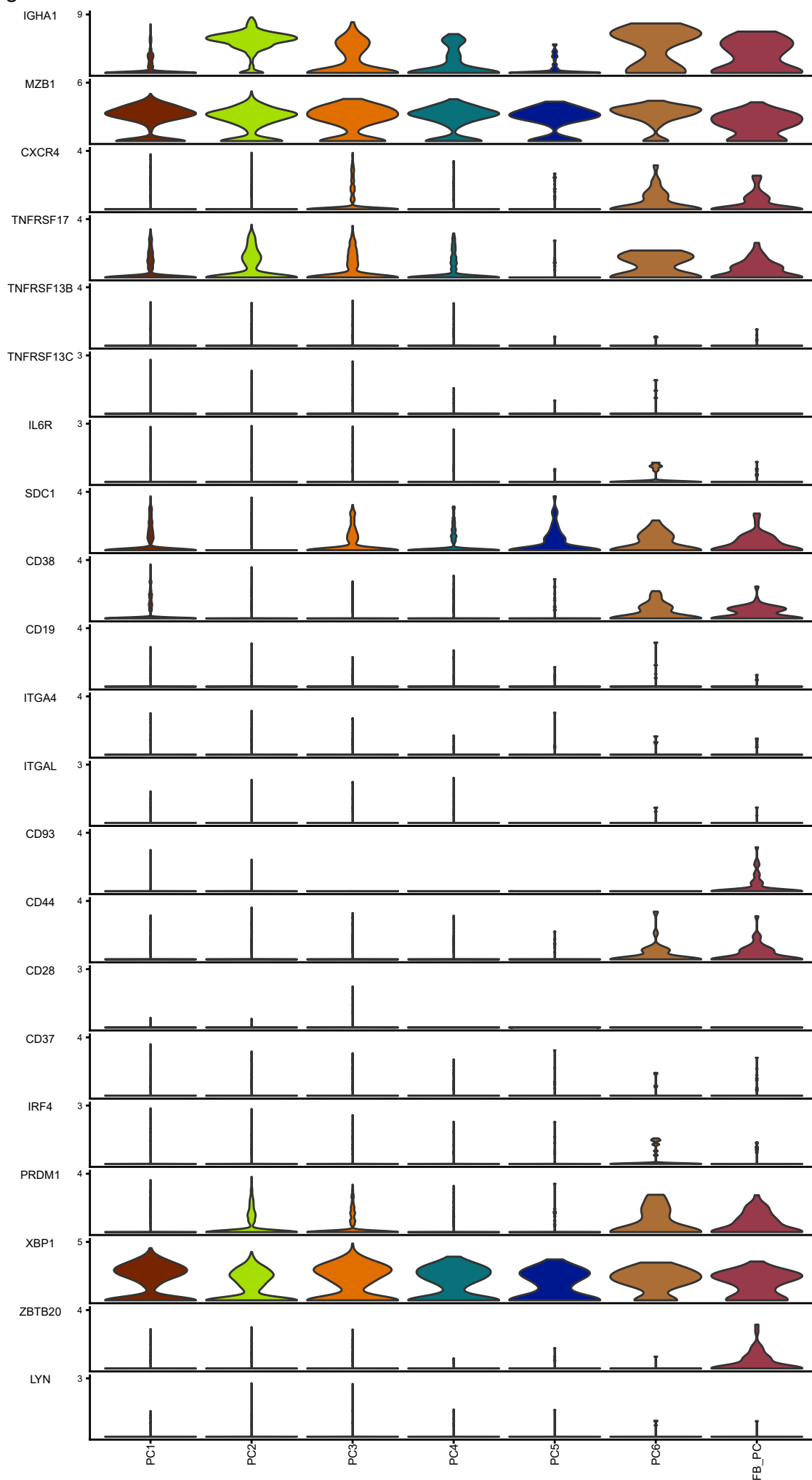

Figure S5

(A)

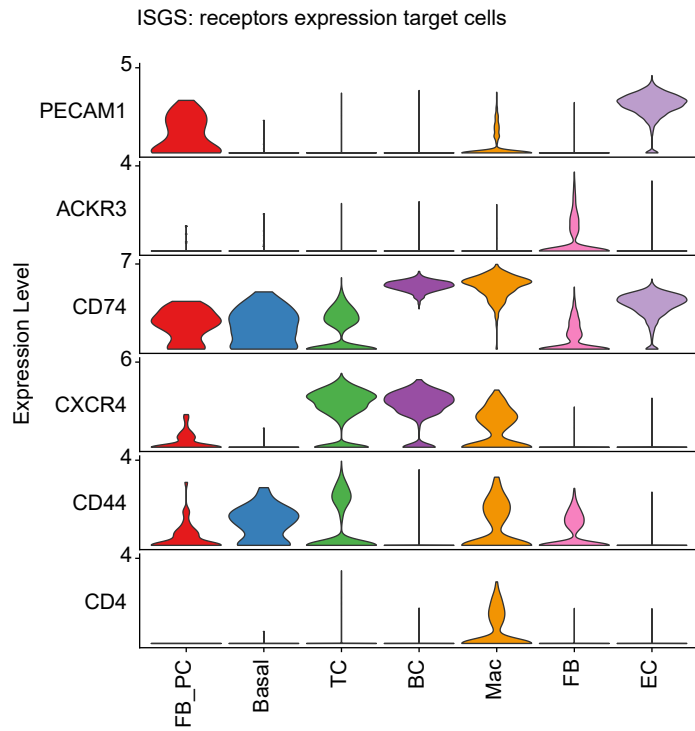

(B)

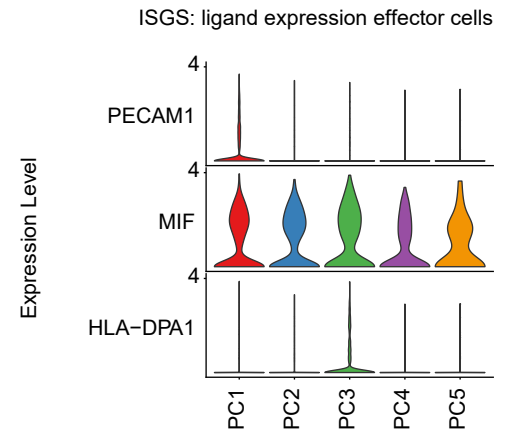

(C)

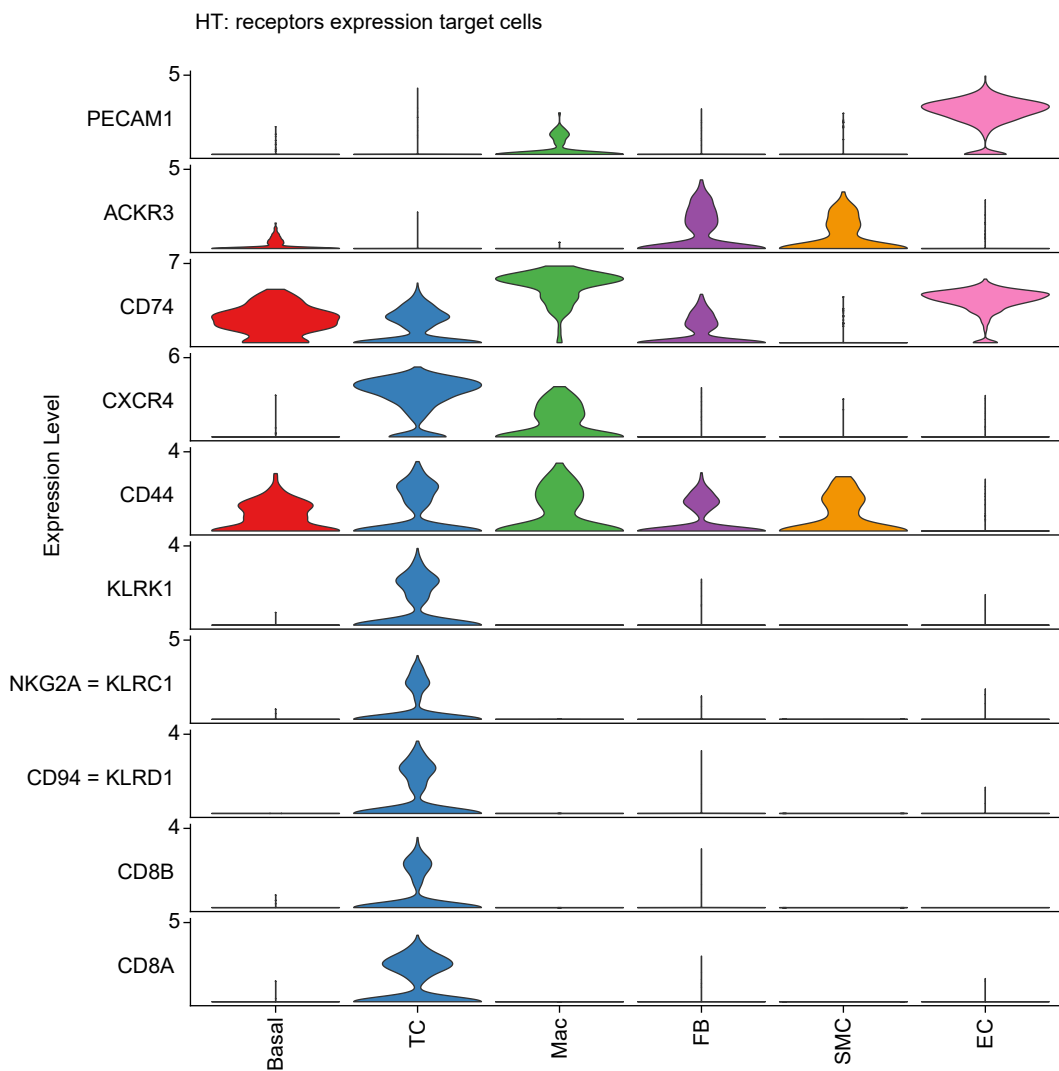

(D)

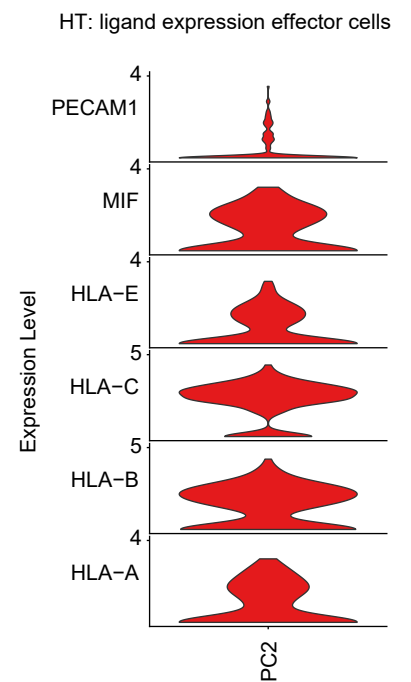

Figure S6

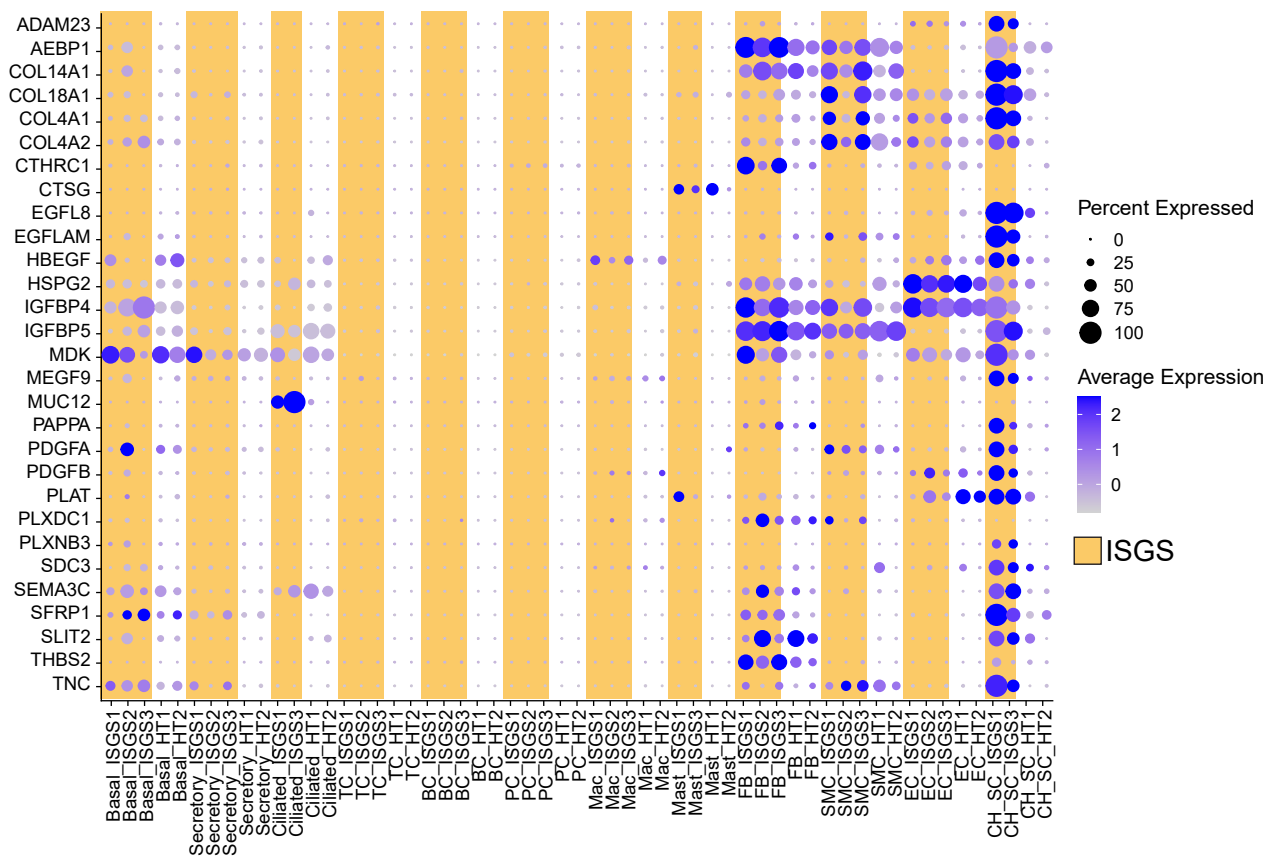

Figure S7

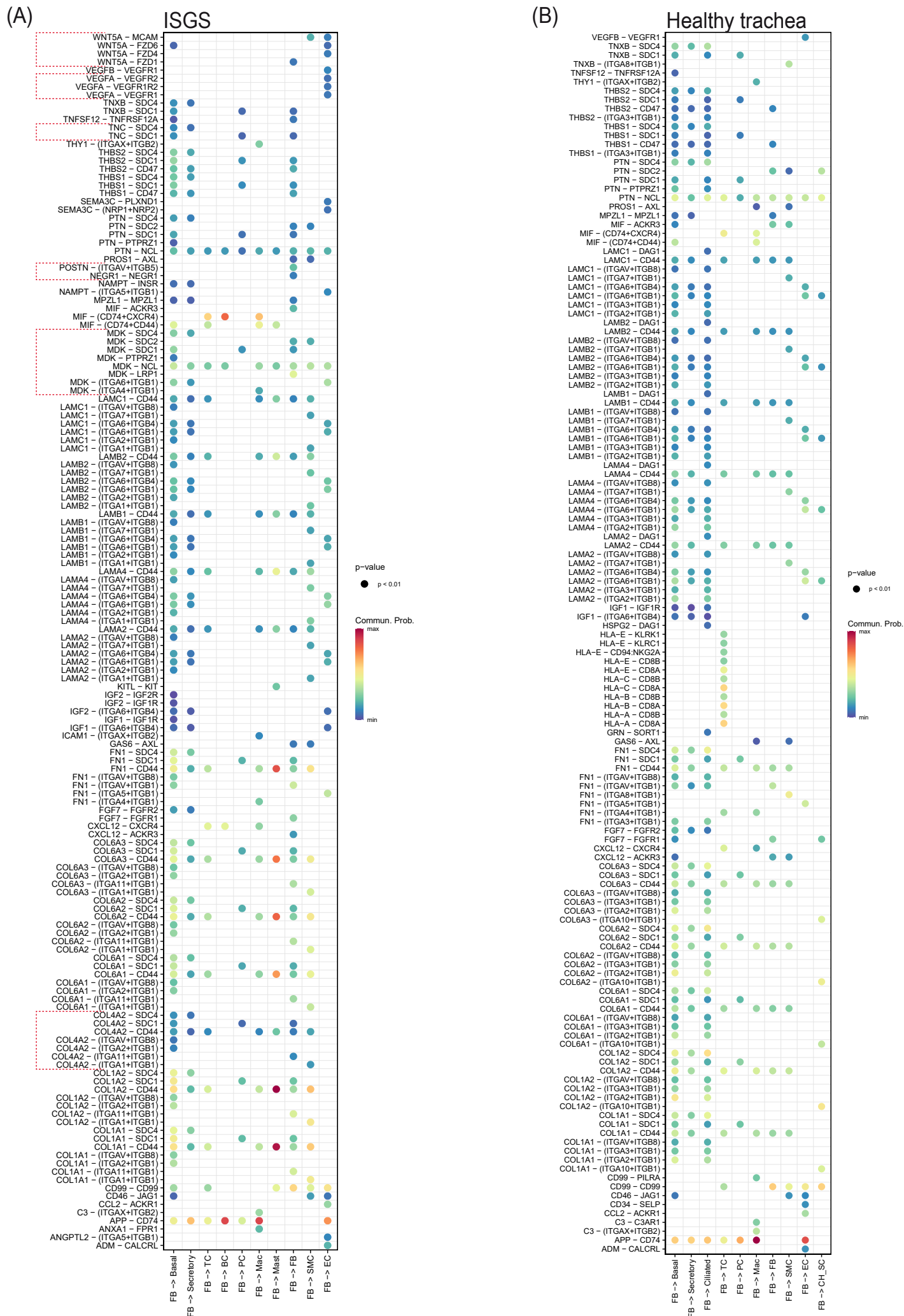

Figure S8

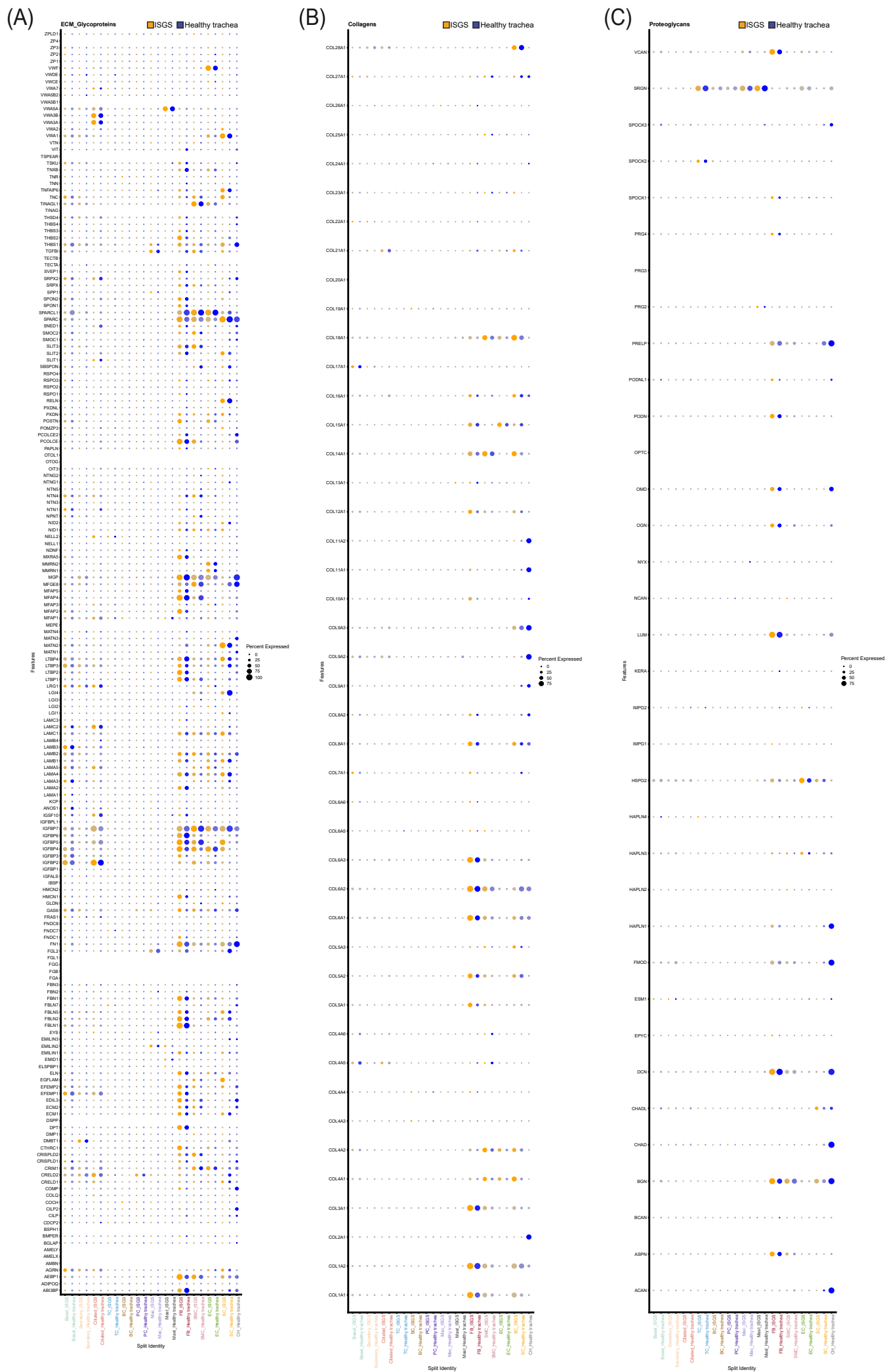

Figure S9

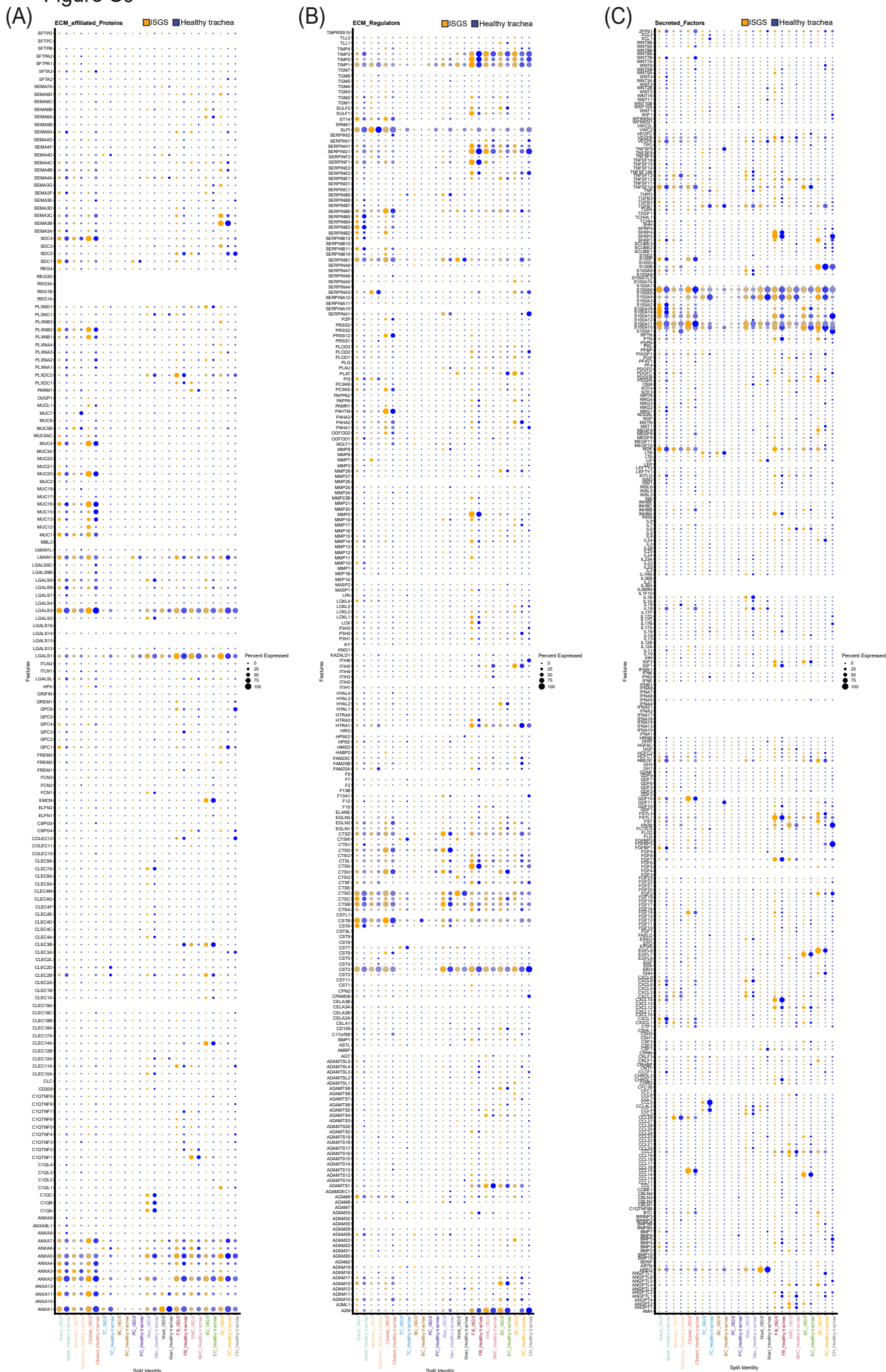

Supplement: Supplementary file 4 [file DataSheet1.PDF]
