# Supplementary material for: Transcriptional profiling sheds light on the fibrotic aspects of idiopathic subglottic tracheal stenosis
Source: Front Cell Dev Biol. 2024 Jul 12;12:1380902. doi: 10.3389/fcell.2024.1380902 (PMC11272577; doi:10.3389/fcell.2024.1380902)
Supplement: Supplementary file 7 [file Image1.pdf]

Legends Supplementary Figures

Figure S1:
UMAP-Plot after integration of all datasets split by donor.
Main UMAP-Plot resulting from integrations of all included scRNAseq datasets split by donor.

Figure S2:
Dotplot of well-known marker genes split by sample
Dotplot depicting expression of well-known marker genes for cluster characterization in all detected cell cluster.

Figure S3:
Dotplot of Top10 upregulated clustermarker split by sample
Dotplot depicting the top 10 upregulated cell type-specific differentially expressed genes. Dot size represents percentage of all cells expressing the gene, color gradient symbolizes average gene expression.

Figure S4:
Transcriptional data allowed no conventional PC annotation
(A) Violinplots depict expression of previously described PC marker, specific for well-characterized PC subtypes. Violin-width shows frequency of cells at the respective expression level.

Figure S5:
Expression comparison of identified receptor and ligand associated genes.
Violin plots depicting expression of cell type-specific identified cell-communication receptor and ligand associated genes in ISGS (A&B) and healthy trachea (C&D).

Figure S6:
Matrix associated condition specific gene regulation.
Dotplot depicting expression of matrix-associated genes by distinct cell types of all donors.

Figure S7:
Potential cell-cell interactions of fibroblasts in ISGS and healthy trachea
Dotplots depicting receptor-ligand couples of FB-subtypes with other cell types in ISGS (A) and healthy trachea tissue (B).

Figure S8:
Expression of ECM-associated glycoproteins, collagens and proteoglycans in cells of healthy trachea and ISGS.
Comparison of glycoproteins (A), collagens (B) and proteoglycans (C) between the cells of healthy trachea and ISGS. (Basal), secretory cells (Secretory), ciliated cells (Ciliated), T-cells (TC), B-cells (BC), plasma cells (PC), macrophages (Mac), mast cells (Mast), fibroblasts (FB), smooth muscle cells (SMC), endothelial cells (EC), Chondrocyte-Schwann cells (CH_SC); Dot-size depicts percentage of cells in a group expressing the gene. Dot color symbolizes average gene expression.

Figure S9:
Expression of ECM-affiliated proteins, ECM regulators and ECM associated secreted factors in cells of healthy trachea and ISGS.
Comparison of ECM-affiliated proteins (A), ECM regulators (B) and ECM-associated secreted factors (C) between the cells of healthy trachea and ISGS. (Basal), secretory cells (Secretory), ciliated cells (Ciliated), T-cells (TC), B-cells (BC), plasma cells (PC), macrophages (Mac), mast cells (Mast), fibroblasts (FB), smooth muscle cells (SMC), endothelial cells (EC), Chondrocyte-Schwann cells (CH_SC); Dot-size depicts percentage of cells in a group expressing the gene. Dot color symbolizes average gene expression.

Figure S1

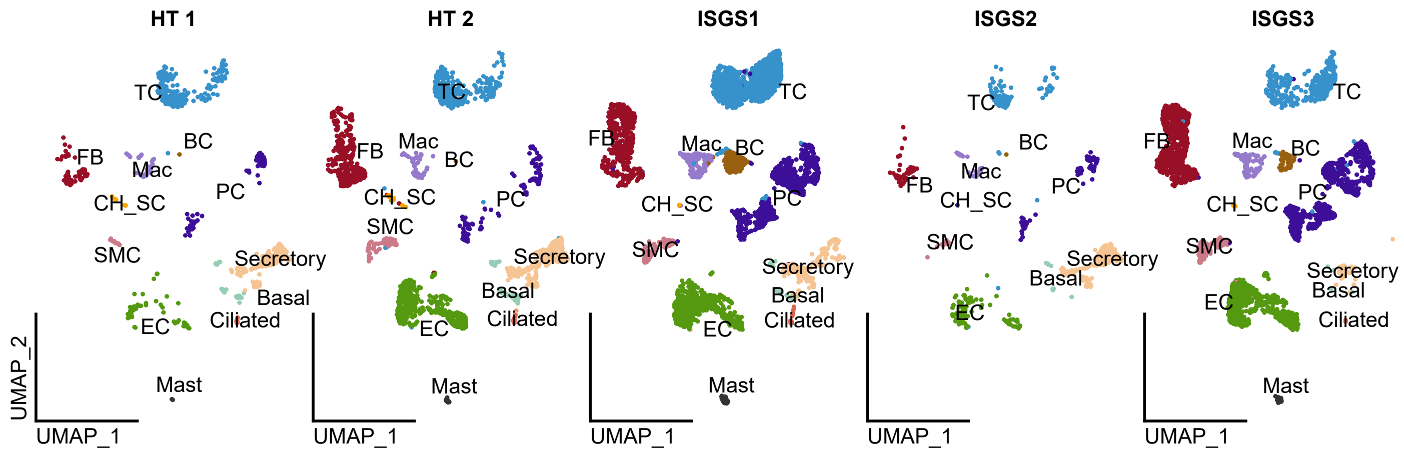
HT 1
HT 2
ISGS1
ISGS2
ISGS3
TC
FB
Mac
BC
CH_SC
PC
SMC
Secretory
Basal
Ciliated
EC
Mast
UMAP_2
UMAP_1

Figure S2

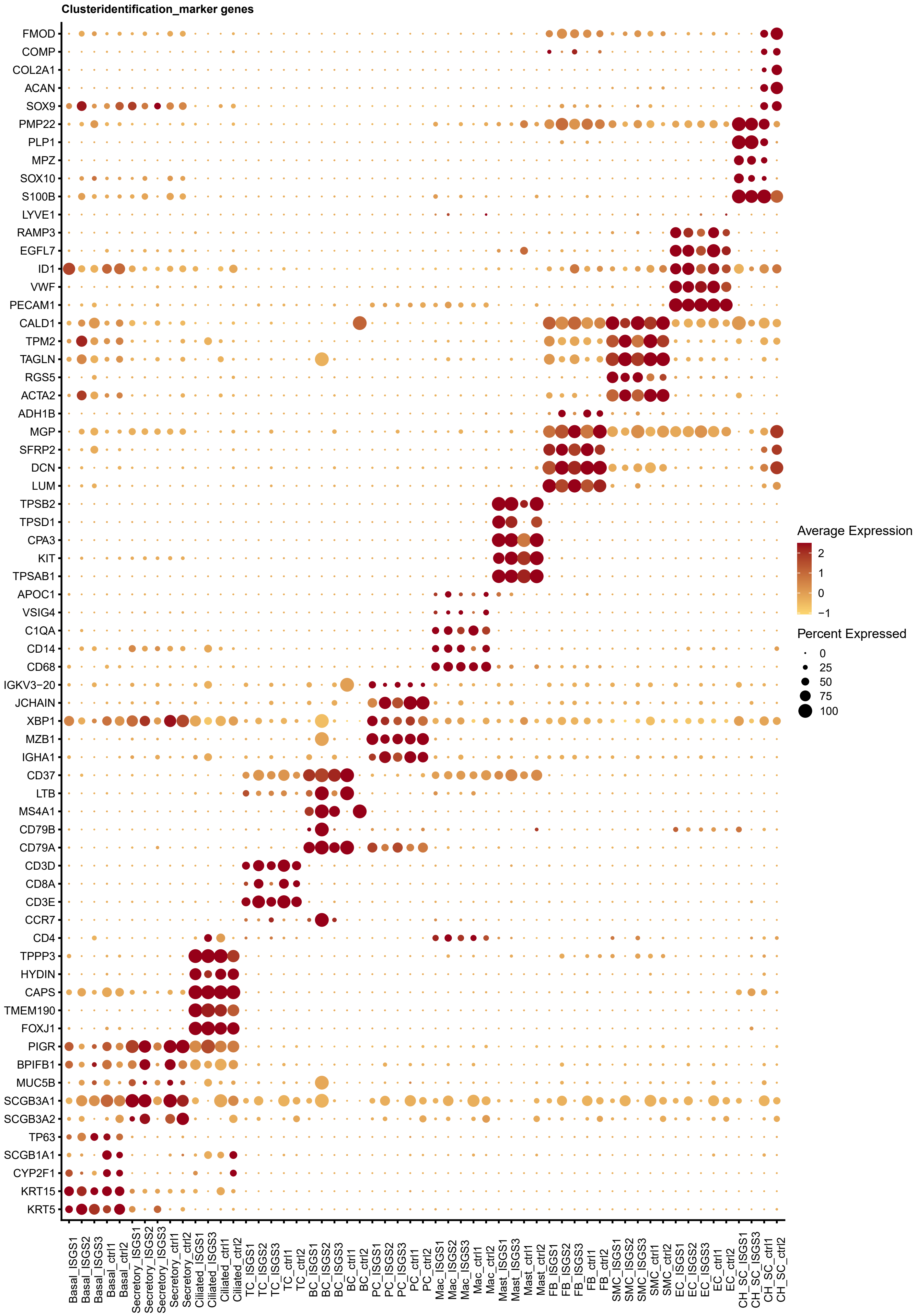
Clusteridentification_marker genes
FMOD
COMP
COL2A1
ACAN
SOX9
PMP22
PLP1
MPZ
SOX10
S100B
LYVE1
RAMP3
EGFL7
ID1
VWF
PECAM1
CALD1
TPM2
TAGLN
RGS5
ACTA2
ADH1B
MGP
SFRP2
DCN
LUM
TPSB2
TPSD1
CPA3
KIT
TPSAB1
APOC1
VSIG4
C1QA
CD14
CD68
IGKV3−20
JCHAIN
XBP1
MZB1
IGHA1
CD37
LTB
MS4A1
CD79B
CD79A
CD3D
CD8A
CD3E
CCR7
CD4
TPPP3
HYDIN
CAPS
TMEM190
FOXJ1
PIGR
BPIFB1
MUC5B
SCGB3A1
SCGB3A2
TP63
SCGB1A1
CYP2F1
KRT15
KRT5
Basal_ISGS1
Basal_ISGS2
Basal_ISGS3
Basal_ctrl1
Basal_ctrl2
Secretory_ISGS1
Secretory_ISGS2
Secretory_ISGS3
Secretory_ctrl1
Secretory_ctrl2
Ciliated_ISGS1
Ciliated_ISGS3
Ciliated_ctrl1
Ciliated_ctrl2
TC_ISGS1
TC_ISGS2
TC_ISGS3
TC_ctrl1
TC_ctrl2
BC_ISGS1
BC_ISGS2
BC_ISGS3
BC_ctrl1
BC_ctrl2
PC_ISGS1
PC_ISGS2
PC_ISGS3
PC_ctrl1
PC_ctrl2
Mac_ISGS1
Mac_ISGS2
Mac_ISGS3
Mac_ctrl1
Mac_ctrl2
Mast_ISGS1
Mast_ISGS3
Mast_ctrl1
Mast_ctrl2
FB_ISGS1
FB_ISGS2
FB_ISGS3
FB_ctrl1
FB_ctrl2
SMC_ISGS1
SMC_ISGS2
SMC_ISGS3
SMC_ctrl1
SMC_ctrl2
EC_ISGS1
EC_ISGS2
EC_ISGS3
EC_ctrl1
EC_ctrl2
CH_SC_ISGS1
CH_SC_ISGS3
CH_SC_ctrl1
CH_SC_ctrl2
Average Expression
2
1
0
−1
Percent Expressed
0
25
50
75
100

Figure S3

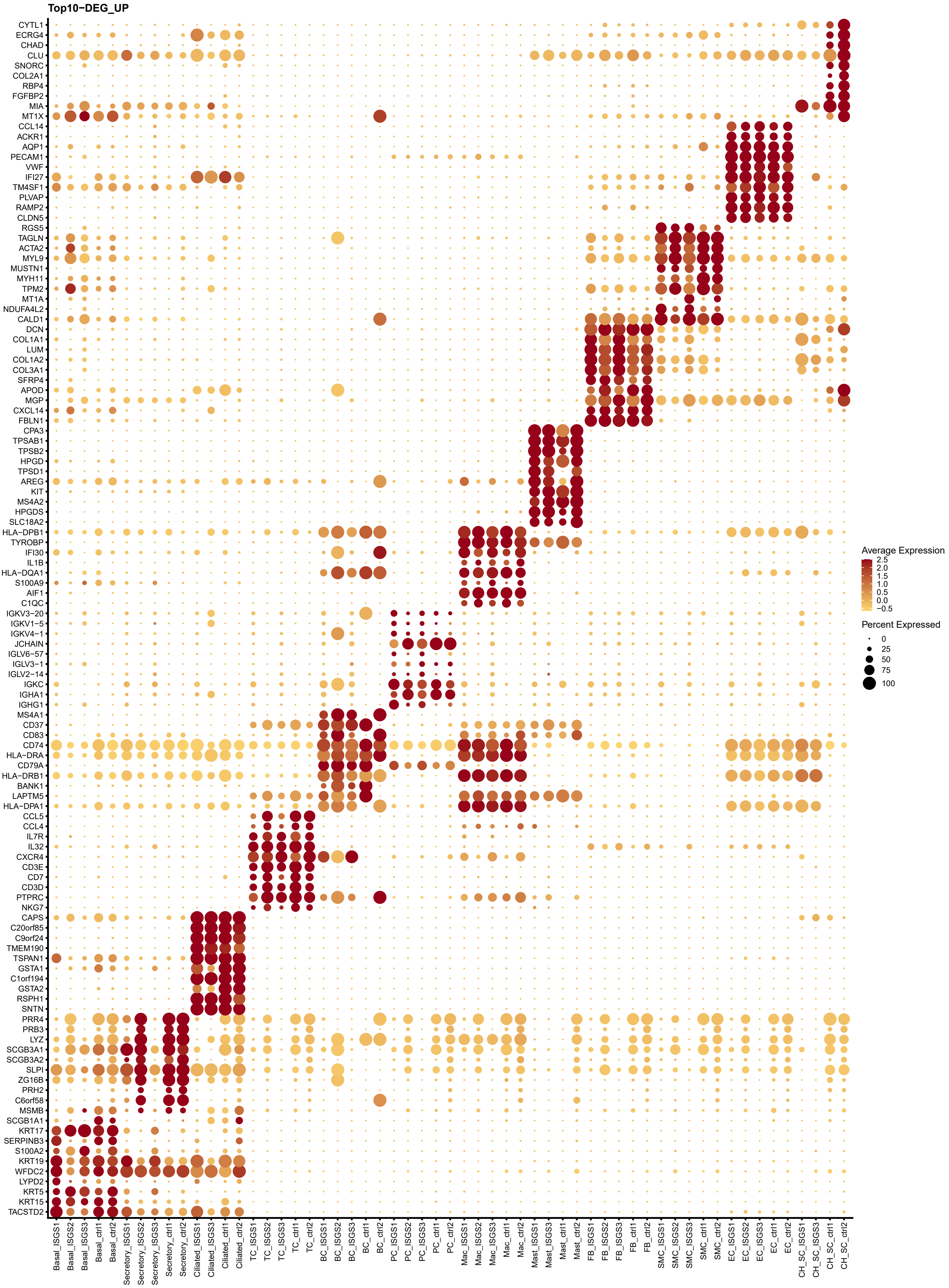
Top10−DEG_UP
CYTL1
ECRG4
CHAD
CLU
SNORC
COL2A1
RBP4
FGFBP2
MIA
MT1X
CCL14
ACKR1
AQP1
PECAM1
VWF
IFI27
TM4SF1
PLVAP
RAMP2
CLDN5
RGS5
TAGLN
ACTA2
MYL9
MUSTN1
MYH11
TPM2
MT1A
NDUFA4L2
CALD1
DCN
COL1A1
LUM
COL1A2
COL3A1
SFRP4
APOD
MGP
CXCL14
FBLN1
CPA3
TPSAB1
TPSB2
HPGD
TPSD1
AREG
KIT
MS4A2
HPGDS
SLC18A2
HLA−DPB1
TYROBP
IFI30
IL1B
HLA−DQA1
S100A9
AIF1
C1QC
IGKV3−20
IGKV1−5
IGKV4−1
JCHAIN
IGLV6−57
IGLV3−1
IGLV2−14
IGKC
IGHA1
IGHG1
MS4A1
CD37
CD83
CD74
HLA−DRA
CD79A
HLA−DRB1
BANK1
LAPTM5
HLA−DPA1
CCL5
CCL4
IL7R
IL32
CXCR4
CD3E
CD7
CD3D
PTPRC
NKG7
CAPS
C20orf85
C9orf24
TMEM190
TSPAN1
GSTA1
C1orf194
GSTA2
RSPH1
SNTN
PRR4
PRB3
LYZ
SCGB3A1
SCGB3A2
SLPI
ZG16B
PRH2
C6orf58
MSMB
SCGB1A1
KRT17
SERPINB3
S100A2
KRT19
WFDC2
LYPD2
KRT5
KRT15
TACSTD2
Basal_ISGS1
Basal_ISGS2
Basal_ISGS3
Basal_ctrl1
Basal_ctrl2
Secretory_ISGS1
Secretory_ISGS2
Secretory_ISGS3
Secretory_ctrl1
Secretory_ctrl2
Ciliated_ISGS1
Ciliated_ISGS3
Ciliated_ctrl1
Ciliated_ctrl2
TC_ISGS1
TC_ISGS2
TC_ISGS3
TC_ctrl1
TC_ctrl2
BC_ISGS1
BC_ISGS2
BC_ISGS3
BC_ctrl1
BC_ctrl2
PC_ISGS1
PC_ISGS2
PC_ISGS3
PC_ctrl1
PC_ctrl2
Mac_ISGS1
Mac_ISGS2
Mac_ISGS3
Mac_ctrl1
Mac_ctrl2
Mast_ISGS1
Mast_ISGS3
Mast_ctrl1
Mast_ctrl2
FB_ISGS1
FB_ISGS2
FB_ISGS3
FB_ctrl1
FB_ctrl2
SMC_ISGS1
SMC_ISGS2
SMC_ISGS3
SMC_ctrl1
SMC_ctrl2
EC_ISGS1
EC_ISGS2
EC_ISGS3
EC_ctrl1
EC_ctrl2
CH_SC_ISGS1
CH_SC_ISGS3
CH_SC_ctrl1
CH_SC_ctrl2
Average Expression
2.5
2.0
1.5
1.0
0.5
0.0
−0.5
Percent Expressed
0
25
50
75
100

Figure S4

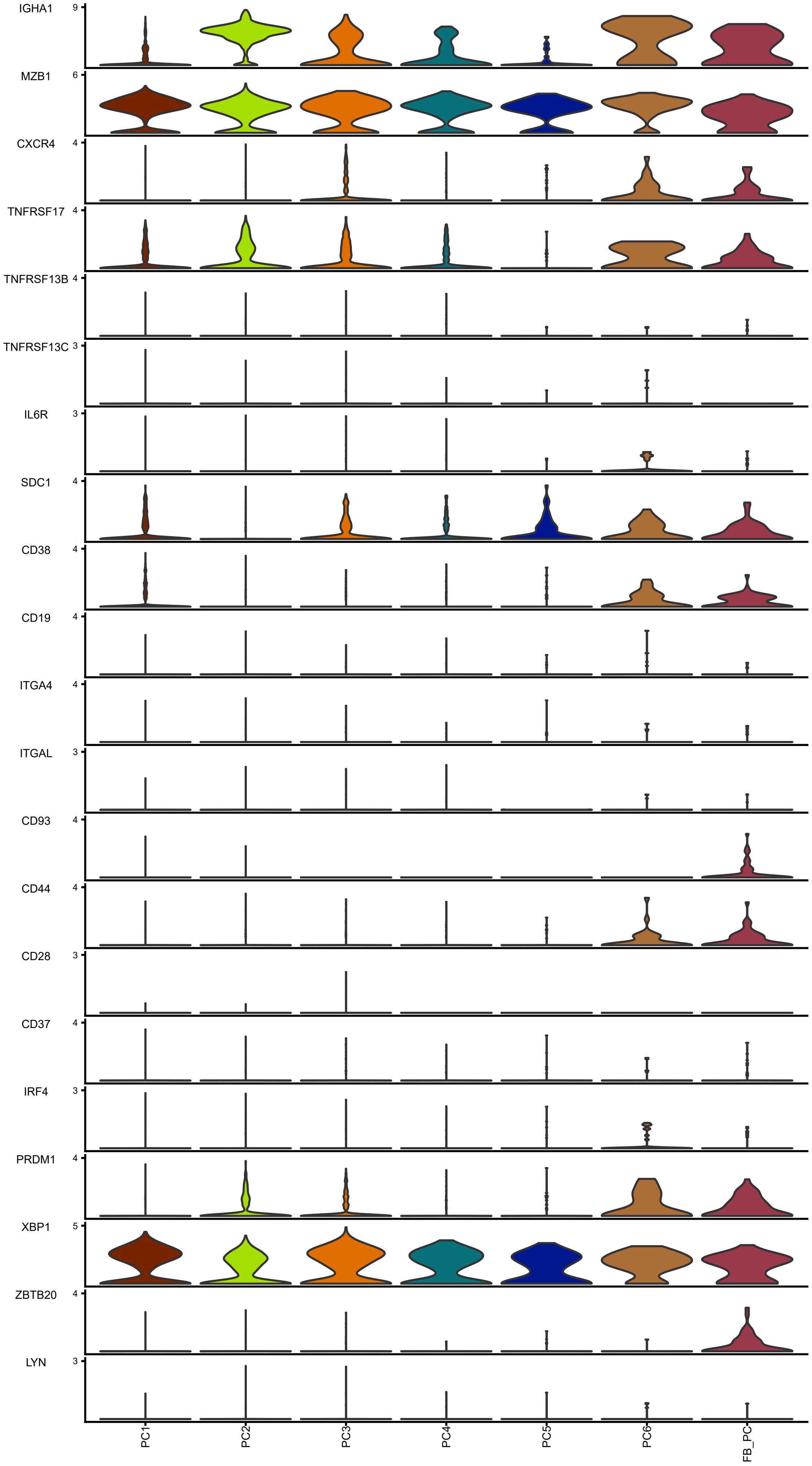
IGHA1
9
MZB1
6
CXCR4
4
TNFRSF17
4
TNFRSF13B
4
TNFRSF13C
3
IL6R
3
SDC1
4
CD38
4
CD19
4
ITGA4
4
ITGAL
3
CD93
4
CD44
4
CD28
3
CD37
4
IRF4
3
PRDM1
4
XBP1
5
ZBTB20
4
LYN
3
PC1
PC2
PC3
PC4
PC5
PC6
FB_PC

Figure S5

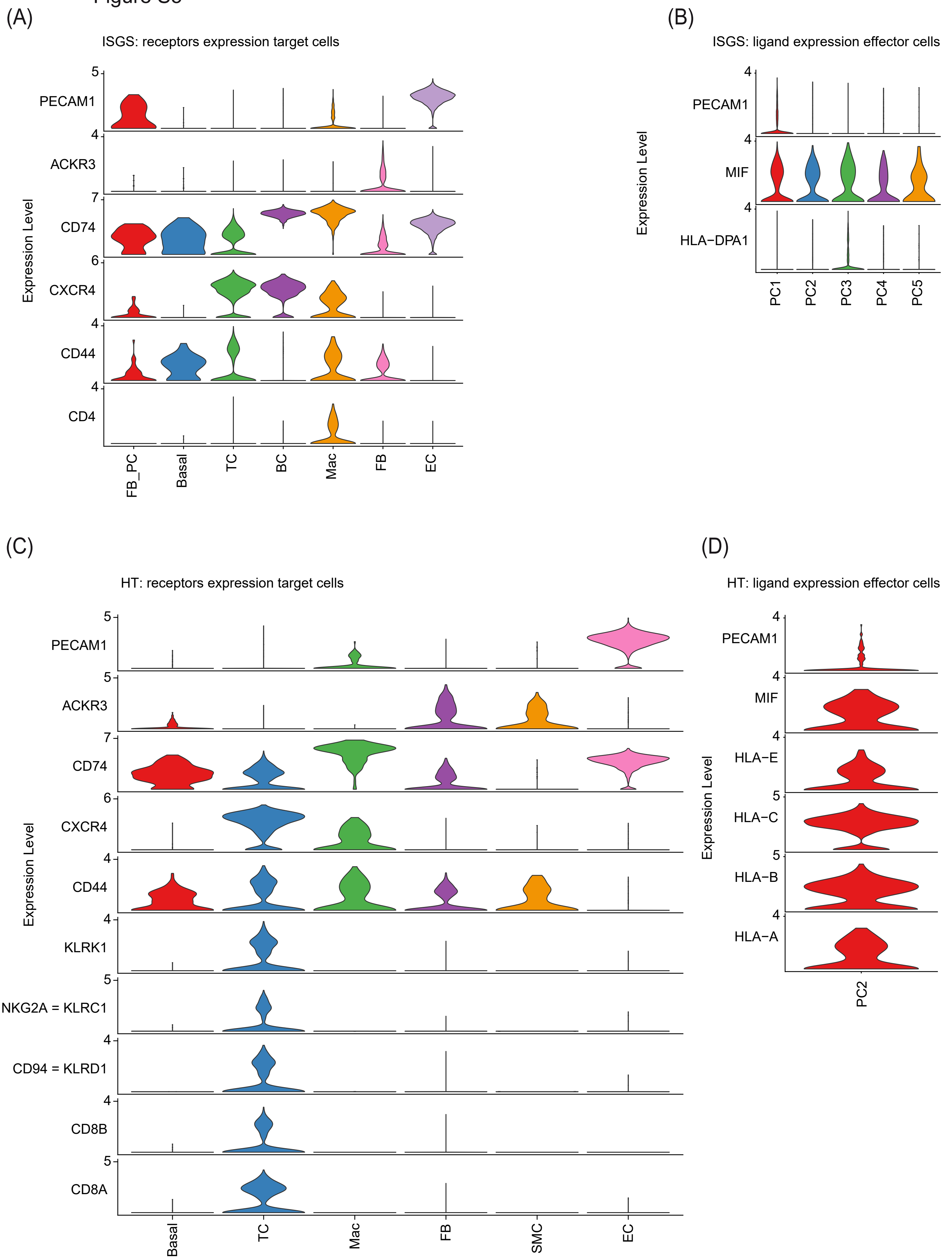

(A)
ISGS: receptors expression target cells
PECAM1
ACKR3
CD74
CXCR4
CD44
CD4
Expression Level
5
4
7
6
4
4
FB_PC
Basal
TC
BC
Mac
FB
EC
(B)
ISGS: ligand expression effector cells
PECAM1
MIF
HLA−DPA1
Expression Level
4
4
4
PC1
PC2
PC3
PC4
PC5
(C)
HT: receptors expression target cells
PECAM1
ACKR3
CD74
CXCR4
CD44
KLRK1
NKG2A = KLRC1
CD94 = KLRD1
CD8B
CD8A
Expression Level
5
5
7
6
4
4
5
4
4
5
Basal
TC
Mac
FB
SMC
EC
(D)
HT: ligand expression effector cells
PECAM1
MIF
HLA−E
HLA−C
HLA−B
HLA−A
Expression Level
4
4
4
5
5
4
PC2

Figure S6

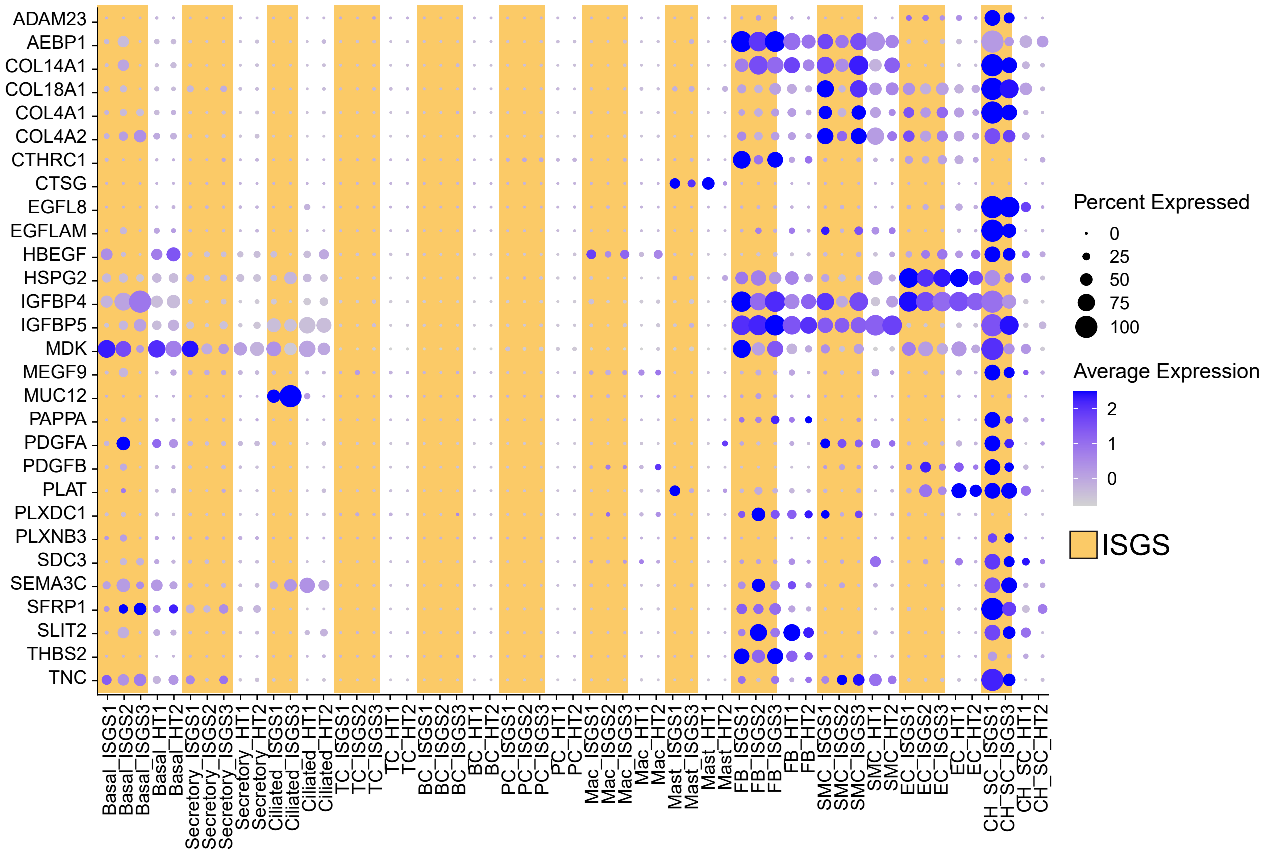
ADAM23
AEBP1
COL14A1
COL18A1
COL4A1
COL4A2
CTHRC1
CTSG
EGFL8
EGFLAM
HBEGF
HSPG2
IGFBP4
IGFBP5
MDK
MEGF9
MUC12
PAPPA
PDGFA
PDGFB
PLAT
PLXDC1
PLXNB3
SDC3
SEMA3C
SFRP1
SLIT2
THBS2
TNC
Basal_ISGS1
Basal_ISGS2
Basal_ISGS3
Basal_HT1
Basal_HT2
Secretory_ISGS1
Secretory_ISGS2
Secretory_ISGS3
Secretory_HT1
Secretory_HT2
Ciliated_ISGS1
Ciliated_ISGS3
Ciliated_HT1
Ciliated_HT2
TC_ISGS1
TC_ISGS2
TC_ISGS3
TC_HT1
TC_HT2
BC_ISGS1
BC_ISGS2
BC_ISGS3
BC_HT1
BC_HT2
PC_ISGS1
PC_ISGS2
PC_ISGS3
PC_HT1
PC_HT2
Mac_ISGS1
Mac_ISGS2
Mac_ISGS3
Mac_HT1
Mac_HT2
Mast_ISGS1
Mast_ISGS3
Mast_HT1
Mast_HT2
FB_ISGS1
FB_ISGS2
FB_ISGS3
FB_HT1
FB_HT2
SMC_ISGS1
SMC_ISGS2
SMC_ISGS3
SMC_HT1
SMC_HT2
EC_ISGS1
EC_ISGS2
EC_ISGS3
EC_HT1
EC_HT2
CH_SC_ISGS1
CH_SC_ISGS3
CH_SC_HT1
CH_SC_HT2
Percent Expressed
0
25
50
75
100
Average Expression
2
1
0
ISGS

Figure S7

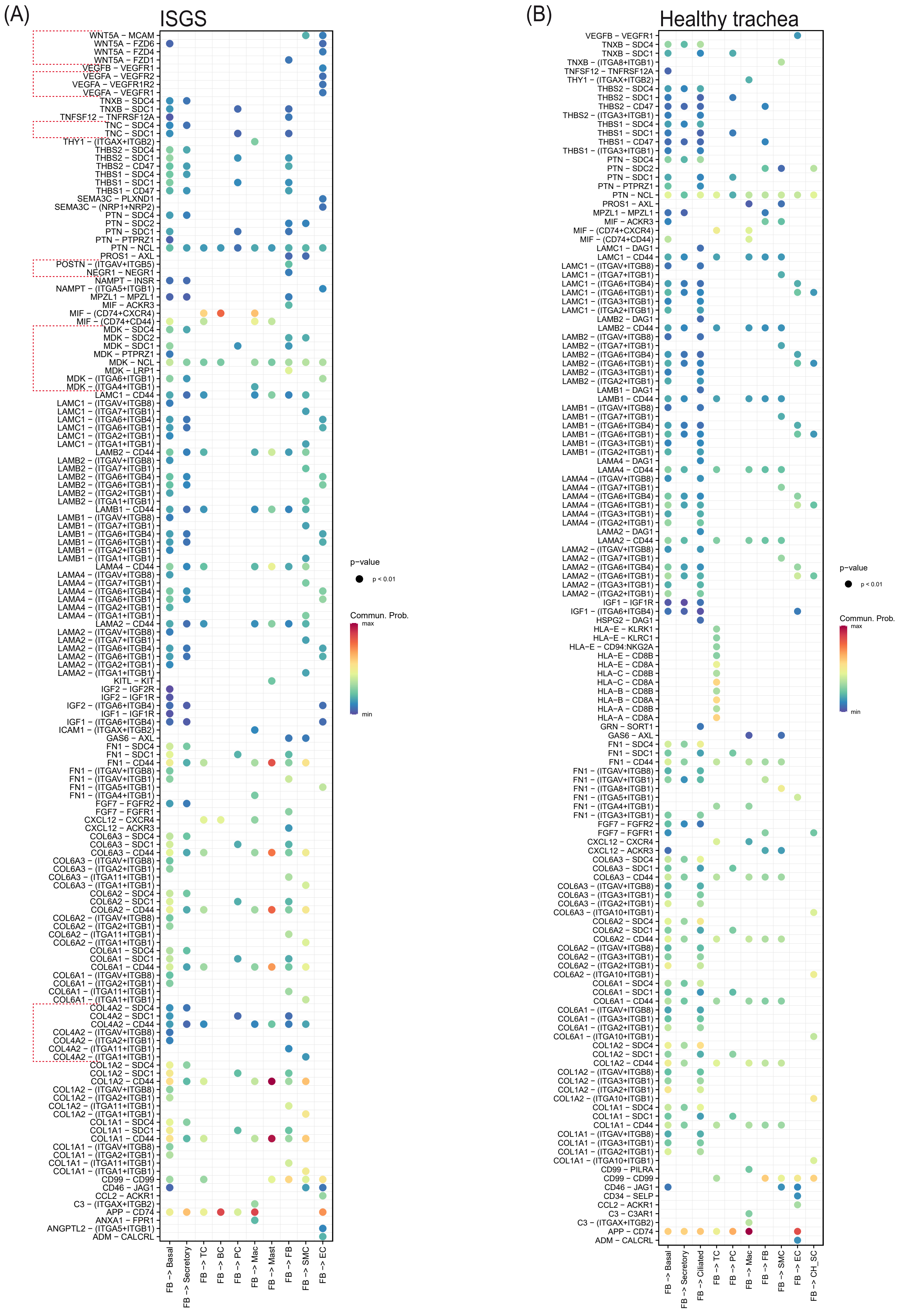
(A)
ISGS
WNT5A – MCAM
WNT5A – FZD6
WNT5A – FZD4
WNT5A – FZD1
VEGFB – VEGFR1
VEGFA – VEGFR2
VEGFA – VEGFR1R2
VEGFA – VEGFR1
TNXB – SDC4
TNXB – SDC1
TNFSF12 – TNFRSF12A
TNC – SDC4
TNC – SDC1
THY1 – (ITGAX+ITGB2)
THBS2 – SDC4
THBS2 – SDC1
THBS2 – CD47
THBS1 – SDC4
THBS1 – SDC1
THBS1 – CD47
SEMA3C – PLXND1
SEMA3C – (NRP1+NRP2)
PTN – SDC4
PTN – SDC2
PTN – SDC1
PTN – PTPRZ1
PTN – NCL
PROS1 – AXL
POSTN – (ITGAV+ITGB5)
NEGR1 – NEGR1
NAMPT – INSR
NAMPT – (ITGA5+ITGB1)
MPZL1 – MPZL1
MIF – ACKR3
MIF – (CD74+CXCR4)
MIF – (CD74+CD44)
MDK – SDC4
MDK – SDC2
MDK – SDC1
MDK – PTPRZ1
MDK – NCL
MDK – LRP1
MDK – (ITGA6+ITGB1)
MDK – (ITGA4+ITGB1)
LAMC1 – CD44
LAMC1 – (ITGAV+ITGB8)
LAMC1 – (ITGA7+ITGB1)
LAMC1 – (ITGA6+ITGB4)
LAMC1 – (ITGA6+ITGB1)
LAMC1 – (ITGA2+ITGB1)
LAMC1 – (ITGA1+ITGB1)
LAMB2 – CD44
LAMB2 – (ITGAV+ITGB8)
LAMB2 – (ITGA7+ITGB1)
LAMB2 – (ITGA6+ITGB4)
LAMB2 – (ITGA6+ITGB1)
LAMB2 – (ITGA2+ITGB1)
LAMB2 – (ITGA1+ITGB1)
LAMB1 – CD44
LAMB1 – (ITGAV+ITGB8)
LAMB1 – (ITGA7+ITGB1)
LAMB1 – (ITGA6+ITGB4)
LAMB1 – (ITGA6+ITGB1)
LAMB1 – (ITGA2+ITGB1)
LAMB1 – (ITGA1+ITGB1)
LAMA4 – CD44
LAMA4 – (ITGAV+ITGB8)
LAMA4 – (ITGA7+ITGB1)
LAMA4 – (ITGA6+ITGB4)
LAMA4 – (ITGA6+ITGB1)
LAMA4 – (ITGA2+ITGB1)
LAMA4 – (ITGA1+ITGB1)
LAMA2 – CD44
LAMA2 – (ITGAV+ITGB8)
LAMA2 – (ITGA7+ITGB1)
LAMA2 – (ITGA6+ITGB4)
LAMA2 – (ITGA6+ITGB1)
LAMA2 – (ITGA2+ITGB1)
LAMA2 – (ITGA1+ITGB1)
KITL – KIT
IGF2 – IGF2R
IGF2 – IGF1R
IGF2 – (ITGA6+ITGB4)
IGF1 – IGF1R
IGF1 – (ITGA6+ITGB4)
ICAM1 – (ITGAX+ITGB2)
GAS6 – AXL
FN1 – SDC4
FN1 – SDC1
FN1 – CD44
FN1 – (ITGAV+ITGB8)
FN1 – (ITGAV+ITGB1)
FN1 – (ITGA5+ITGB1)
FN1 – (ITGA4+ITGB1)
FGF7 – FGFR2
FGF7 – FGFR1
CXCL12 – CXCR4
CXCL12 – ACKR3
COL6A3 – SDC4
COL6A3 – SDC1
COL6A3 – CD44
COL6A3 – (ITGAV+ITGB8)
COL6A3 – (ITGA2+ITGB1)
COL6A3 – (ITGA11+ITGB1)
COL6A3 – (ITGA1+ITGB1)
COL6A2 – SDC4
COL6A2 – SDC1
COL6A2 – CD44
COL6A2 – (ITGAV+ITGB8)
COL6A2 – (ITGA2+ITGB1)
COL6A2 – (ITGA11+ITGB1)
COL6A2 – (ITGA1+ITGB1)
COL6A1 – SDC4
COL6A1 – SDC1
COL6A1 – CD44
COL6A1 – (ITGAV+ITGB8)
COL6A1 – (ITGA2+ITGB1)
COL6A1 – (ITGA11+ITGB1)
COL6A1 – (ITGA1+ITGB1)
COL4A2 – SDC4
COL4A2 – SDC1
COL4A2 – CD44
COL4A2 – (ITGAV+ITGB8)
COL4A2 – (ITGA2+ITGB1)
COL4A2 – (ITGA11+ITGB1)
COL4A2 – (ITGA1+ITGB1)
COL1A2 – SDC4
COL1A2 – SDC1
COL1A2 – CD44
COL1A2 – (ITGAV+ITGB8)
COL1A2 – (ITGA2+ITGB1)
COL1A2 – (ITGA11+ITGB1)
COL1A2 – (ITGA1+ITGB1)
COL1A1 – SDC4
COL1A1 – SDC1
COL1A1 – CD44
COL1A1 – (ITGAV+ITGB8)
COL1A1 – (ITGA2+ITGB1)
COL1A1 – (ITGA11+ITGB1)
COL1A1 – (ITGA1+ITGB1)
CD99 – CD99
CD46 – JAG1
CCL2 – ACKR1
C3 – (ITGAX+ITGB2)
APP – CD74
ANXA1 – FPR1
ANGPTL2 – (ITGA5+ITGB1)
ADM – CALCRL
FB –> Basal
FB –> Secretory
FB –> TC
FB –> BC
FB –> PC
FB –> Mac
FB –> Mast
FB –> FB
FB –> SMC
FB –> EC
p–value
p < 0.01
Commun. Prob.
max
min
(B)
Healthy trachea
VEGFB – VEGFR1
TNXB – SDC4
TNXB – SDC1
TNXB – (ITGA8+ITGB1)
TNFSF12 – TNFRSF12A
THY1 – (ITGAX+ITGB2)
THBS2 – SDC4
THBS2 – SDC1
THBS2 – CD47
THBS2 – (ITGA3+ITGB1)
THBS1 – SDC4
THBS1 – SDC1
THBS1 – CD47
THBS1 – (ITGA3+ITGB1)
PTN – SDC4
PTN – SDC2
PTN – SDC1
PTN – PTPRZ1
PTN – NCL
PROS1 – AXL
MPZL1 – MPZL1
MIF – ACKR3
MIF – (CD74+CXCR4)
MIF – (CD74+CD44)
LAMC1 – DAG1
LAMC1 – CD44
LAMC1 – (ITGAV+ITGB8)
LAMC1 – (ITGA7+ITGB1)
LAMC1 – (ITGA6+ITGB4)
LAMC1 – (ITGA6+ITGB1)
LAMC1 – (ITGA3+ITGB1)
LAMC1 – (ITGA2+ITGB1)
LAMB2 – DAG1
LAMB2 – CD44
LAMB2 – (ITGAV+ITGB8)
LAMB2 – (ITGA7+ITGB1)
LAMB2 – (ITGA6+ITGB4)
LAMB2 – (ITGA6+ITGB1)
LAMB2 – (ITGA3+ITGB1)
LAMB2 – (ITGA2+ITGB1)
LAMB1 – DAG1
LAMB1 – CD44
LAMB1 – (ITGAV+ITGB8)
LAMB1 – (ITGA7+ITGB1)
LAMB1 – (ITGA6+ITGB4)
LAMB1 – (ITGA6+ITGB1)
LAMB1 – (ITGA3+ITGB1)
LAMB1 – (ITGA2+ITGB1)
LAMA4 – DAG1
LAMA4 – CD44
LAMA4 – (ITGAV+ITGB8)
LAMA4 – (ITGA7+ITGB1)
LAMA4 – (ITGA6+ITGB4)
LAMA4 – (ITGA6+ITGB1)
LAMA4 – (ITGA3+ITGB1)
LAMA4 – (ITGA2+ITGB1)
LAMA2 – DAG1
LAMA2 – CD44
LAMA2 – (ITGAV+ITGB8)
LAMA2 – (ITGA7+ITGB1)
LAMA2 – (ITGA6+ITGB4)
LAMA2 – (ITGA6+ITGB1)
LAMA2 – (ITGA3+ITGB1)
LAMA2 – (ITGA2+ITGB1)
IGF1 – IGF1R
IGF1 – (ITGA6+ITGB4)
HSPG2 – DAG1
HLA–E – KLRK1
HLA–E – KLRC1
HLA–E – CD94:NKG2A
HLA–E – CD8B
HLA–E – CD8A
HLA–C – CD8B
HLA–C – CD8A
HLA–B – CD8B
HLA–B – CD8A
HLA–A – CD8B
HLA–A – CD8A
GRN – SORT1
GAS6 – AXL
FN1 – SDC4
FN1 – SDC1
FN1 – CD44
FN1 – (ITGAV+ITGB8)
FN1 – (ITGAV+ITGB1)
FN1 – (ITGA8+ITGB1)
FN1 – (ITGA5+ITGB1)
FN1 – (ITGA4+ITGB1)
FN1 – (ITGA3+ITGB1)
FGF7 – FGFR2
FGF7 – FGFR1
CXCL12 – CXCR4
CXCL12 – ACKR3
COL6A3 – SDC4
COL6A3 – SDC1
COL6A3 – CD44
COL6A3 – (ITGAV+ITGB8)
COL6A3 – (ITGA3+ITGB1)
COL6A3 – (ITGA2+ITGB1)
COL6A3 – (ITGA10+ITGB1)
COL6A2 – SDC4
COL6A2 – SDC1
COL6A2 – CD44
COL6A2 – (ITGAV+ITGB8)
COL6A2 – (ITGA3+ITGB1)
COL6A2 – (ITGA2+ITGB1)
COL6A2 – (ITGA10+ITGB1)
COL6A1 – SDC4
COL6A1 – SDC1
COL6A1 – CD44
COL6A1 – (ITGAV+ITGB8)
COL6A1 – (ITGA3+ITGB1)
COL6A1 – (ITGA2+ITGB1)
COL6A1 – (ITGA10+ITGB1)
COL1A2 – SDC4
COL1A2 – SDC1
COL1A2 – CD44
COL1A2 – (ITGAV+ITGB8)
COL1A2 – (ITGA3+ITGB1)
COL1A2 – (ITGA2+ITGB1)
COL1A2 – (ITGA10+ITGB1)
COL1A1 – SDC4
COL1A1 – SDC1
COL1A1 – CD44
COL1A1 – (ITGAV+ITGB8)
COL1A1 – (ITGA3+ITGB1)
COL1A1 – (ITGA2+ITGB1)
COL1A1 – (ITGA10+ITGB1)
CD99 – PILRA
CD99 – CD99
CD46 – JAG1
CD34 – SELP
CCL2 – ACKR1
C3 – C3AR1
C3 – (ITGAX+ITGB2)
APP – CD74
ADM – CALCRL
FB –> Basal
FB –> Secretory
FB –> Ciliated
FB –> TC
FB –> PC
FB –> Mac
FB –> FB
FB –> SMC
FB –> EC
FB –> CH_SC
p–value
p < 0.01
Commun. Prob.
max
min

Figure S8

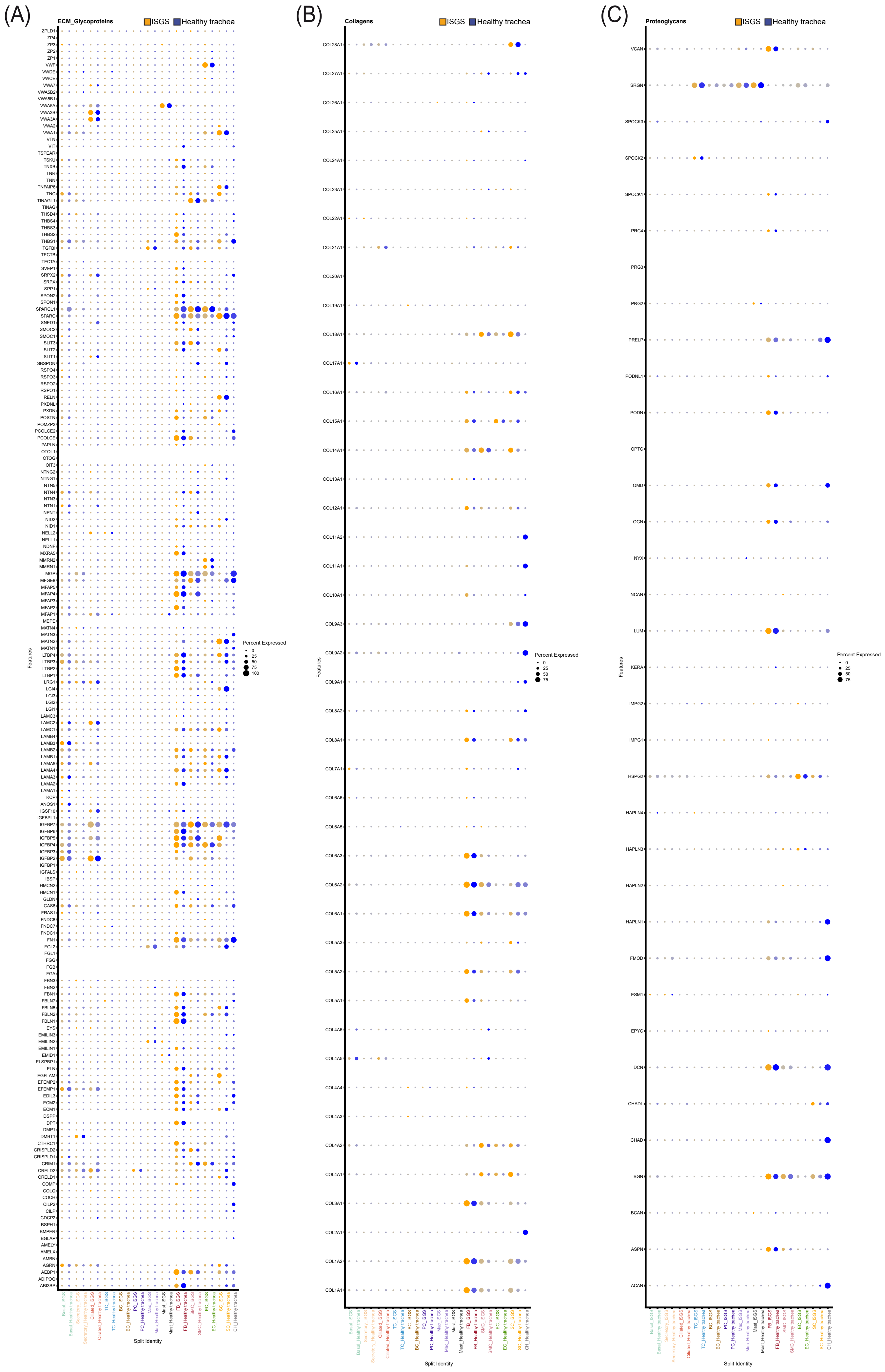

(A)
ECM_Glycoproteins
ISGS
Healthy trachea
Features
Percent Expressed
0
25
50
75
100
Split Identity
(B)
Collagens
ISGS
Healthy trachea
Features
Percent Expressed
0
25
50
75
Split Identity
(C)
Proteoglycans
ISGS
Healthy trachea
Features
Percent Expressed
0
25
50
75
Split Identity

Figure S9

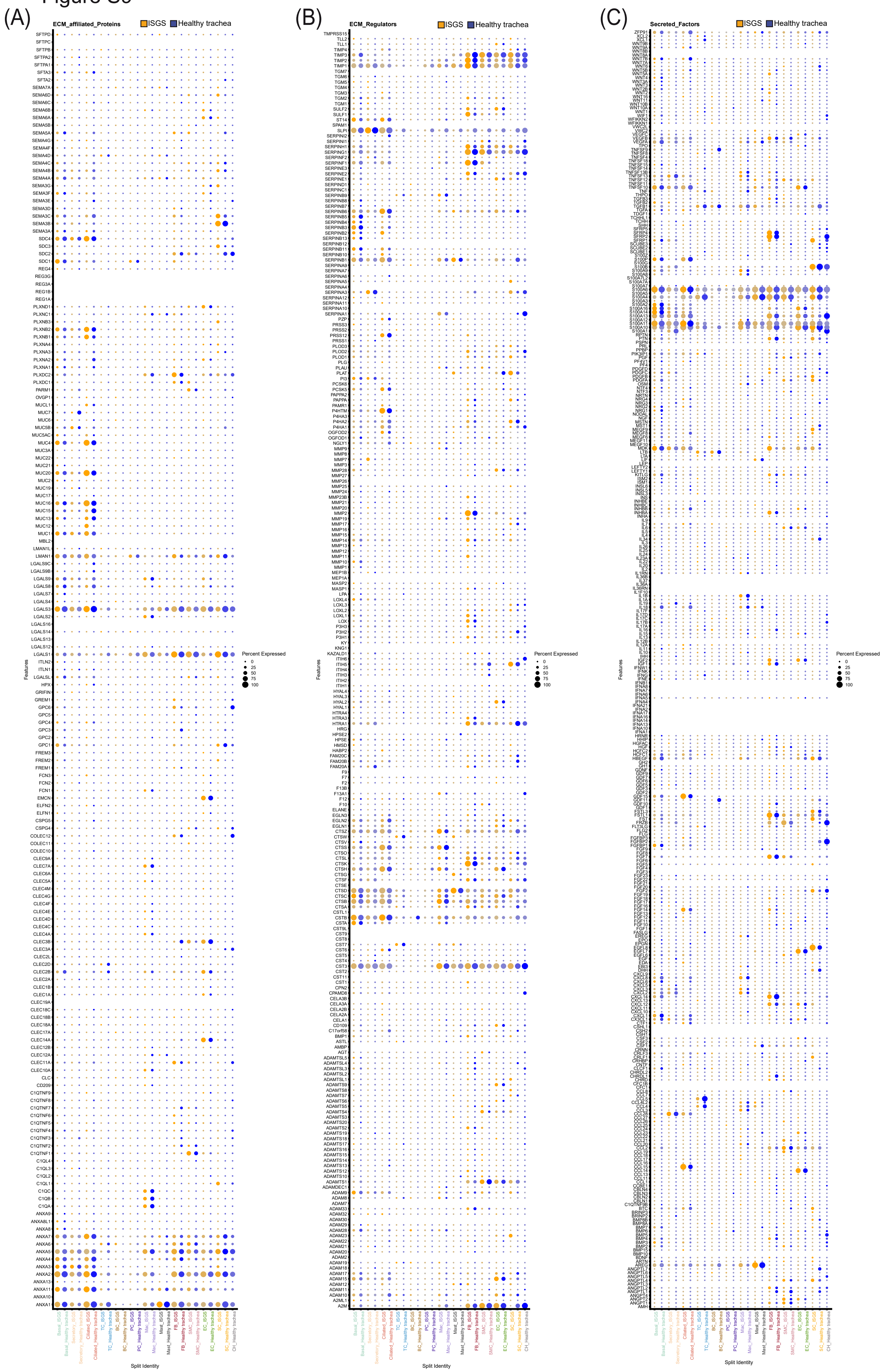
(A)
ECM_affiliated_Proteins
ISGS
Healthy trachea
(B)
ECM_Regulators
ISGS
Healthy trachea
(C)
Secreted_Factors
ISGS
Healthy trachea
Features
Split Identity
Percent Expressed
0
25
50
75
100
